# Collagen-Binding Nanoparticles for Paclitaxel Encapsulation and Breast Cancer Treatment

**DOI:** 10.1021/acsbiomaterials.3c01332

**Published:** 2023-11-20

**Authors:** Julia Sapienza Passos, Luciana B. Lopes, Alyssa Panitch

**Affiliations:** † Wallace H. Coulter Department of Biomedical Engineering, Georgia Institute of Technology and Emory University, Atlanta, Georgia 30332, United States; ‡ Department of Pharmacology, Institute of Biomedical Sciences, University of Sao Paulo, Sao Paulo, SP 05508-000, Brazil

**Keywords:** breast cancer, paclitaxel, lipid-polymeric nanoparticles, poly(*N*-isopropylacrylamide) (pNIPAM), collagen binding peptide

## Abstract

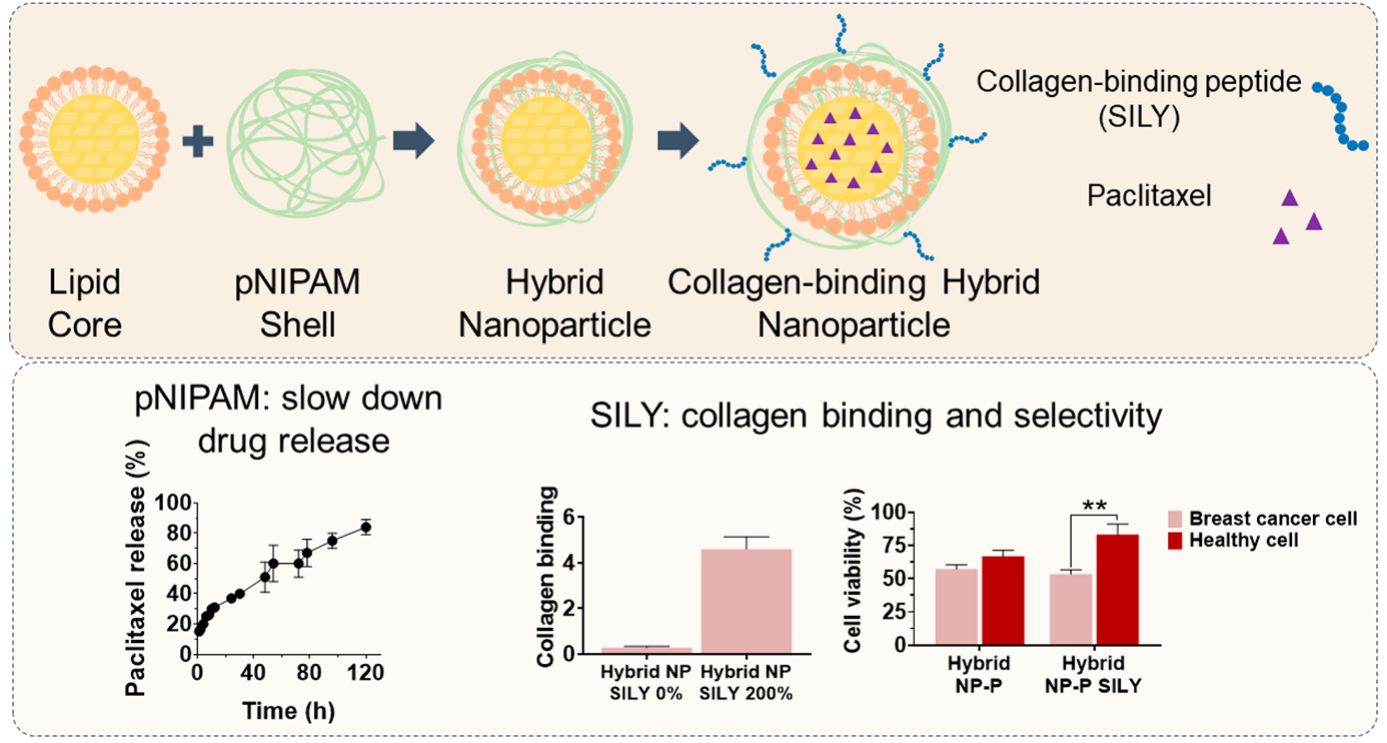

In this study, we developed a novel hybrid collagen-binding nanocarrier for potential intraductal administration and local breast cancer treatment. The particles were formed by the encapsulation of nanostructured lipid carriers (NLCs) containing the cytotoxic drug paclitaxel within a shell of poly(*N*-isopropylacrylamide) (pNIPAM), and were functionalized with SILY, a peptide that binds to collagen type I (which is overexpressed in the mammary tumor microenvironment) to improve local retention and selectivity. The encapsulation of the NLCs in the pNIPAM shell increased nanoparticle size by approximately 140 nm, and after purification, a homogeneous system of hybrid nanoparticles (∼96%) was obtained. The nanoparticles exhibited high loading efficiency (<76%) and were capable of prolonging paclitaxel release for up to 120 h. SILY-modified nanoparticles showed the ability to bind to collagen-coated surfaces and naturally elaborated collagen. Hybrid nanoparticles presented cytotoxicity up to 3.7-fold higher than pNIPAM-only nanoparticles on mammary tumor cells cultured in monolayers. In spheroids, the increase in cytotoxicity was up to 1.8-fold. Compared to lipid nanoparticles, the hybrid nanoparticle modified with SILY increased the viability of nontumor breast cells by up to 1.59-fold in a coculture model, suggesting the effectiveness and safety of the system.

## Introduction

Breast cancer is the most frequently diagnosed cancer worldwide, with over 2.3 million new cases and 685,000 deaths in 2020 alone.^1^ Approximately 20–25% of breast cancers are classified as ductal carcinoma *in situ* (DCIS), which is characterized by the proliferation of neoplastic luminal cells that are confined to the mammary ductolobular system.^2,3^ Considering that most breast cancers originate in the ductal epithelium, intraductal therapy, in which a drug is administered through the nipple into the breast ductal system, allows drug delivery directly to the lesions, increasing local exposure, preventing the development of invasive forms of the disease, and decreasing the incidence of adverse effects resulting from systemic exposure to drugs.^4−6^ The intraductal administration of drugs has been demonstrated to be safe and feasible in several preclinical and clinical studies for different cytotoxic agents.^4,6−9^ Intraductally administered agents were able not only to reduce the size of pre-established tumors but also to prevent the appearance of new tumors.^5^ Thus, it can offer an alternative treatment option for low-grade DCIS and other types of pretumor lesions, for neoadjuvant treatment, and for the delivery of chemopreventive agents.^5,10−13^

Despite its promises, this mode of administration is not trivial and has been described to require training for ductal identification and cannulation and to avoid ductal rupture, especially in DCIS-filled ducts. To enable its successful translation, our group and others have proposed the use of nanocarriers to improve targeting and selectivity and prolong tissue retention to reduce the frequency of administration.^7−9,14,15^ Size, surface modifications, and properties of the encapsulated compound were demonstrated to impact ductal retention and are important features to guide formulation design for this administration route.^4,8,14^

In this study, we propose the development of a hybrid nanocarrier consisting of a nanostructured lipid carrier core with a poly(*N*-isopropylacrylamide) (pNIPAM) shell, aiming to combine the advantages of lipid and polymeric systems. The lipid core allows solubilization and encapsulation of lipophilic drugs (such as paclitaxel), while the polymeric (pNIPAM) shell facilitates surface functionalization and responsiveness to physiological stimuli.^4,16−18^

pNIPAM is a thermoresponsive polymer that has been extensively studied for use in drug delivery systems, in part because it undergoes phase transition around 33 °C (LCST, lower critical solution temperature).^19−24^ At physiological temperatures, pNIPAM undergoes hydrophobic collapse, allowing a sustained release of encapsulated compounds while providing protection from physicochemical degradation. Additionally, pNIPAM can be co-polymerized with co-monomers containing functional groups that allow chemical modification with targeting ligands.^19,20^ To increase nanoparticle functionality, the pNIPAM shell surface was modified with collagen type I-binding peptide (RRANAALKAGELYKSILYGC, abbreviated SILY).^23−25^ Collagen targeting can be justified by the fact that the mammary extracellular matrix undergoes changes during the process of carcinogenesis that include the overexpression of type I collagen.^26−29^ Thus, collagen can potentially be a better target in breast cancer therapy than surface markers and receptors of the neoplastic cells, which may vary according to the tumor type,^28^ leading to prolonged retention at the site of action after intraductal administration and increased selectivity to tumor cells.

In this study, hybrid nanoparticles were produced, and paclitaxel release kinetics, collagen binding, cell uptake mechanisms, and cytotoxicity were assessed and compared to polymeric and lipid nanoparticles (pNIPAM only, referred to here as pNIPAM NPs, and nanostructured lipid carriers (NLCs)). The developed nanoparticulate systems were characterized for their size distribution, zeta potential, morphology, drug encapsulation and release, cell uptake mechanism, and cytotoxic effects on 2D and 3D breast cancer cell models. To further characterize the ability of the nanoparticles to promote tumor targeting, we evaluated the collagen-binding affinity of the different formulations and whether this binding promoted cytotoxic effects, preferably on the target cells. Currently, no hybrid nanoparticles designed for intraductal delivery of cytotoxic drugs have been described in the literature. With this study, we aim to fill this gap and contribute with a strategy for the treatment of DCIS that is effective and limits systemic exposure to the drug.

## Results

### Nanoparticles Development and Characterization

NLCs, employed as cores for the hybrid nanoparticles, were obtained by a fusion emulsification technique.^30^ The development of hybrid nanoparticles started with the optimization of the synthesis temperature to ensure that the NLC was stable at temperatures necessary for the pNIPAM shell synthesis. The NLCs were incubated for 4 h at 50, 60, and 70 °C and characterized by dynamic light scattering (Figure S1, NLC). NLCs heated to 70 °C showed a significant reduction in mean diameter compared to those maintained at room temperature (−75.6% and −40.7% compared to unloaded and paclitaxel-loaded NLCs, respectively). NLCs heated to 50 and 60 °C showed slight reductions in the average diameter, although these were not significant, proving lower temperatures to be more suitable for the synthesis of hybrid core–shell nanoparticles to maintain the approximate characteristics of the lipid core. Similar results were obtained for NLCs loaded with paclitaxel (Figure S1, NLC-P).

Next, we evaluated pNIPAM (only) nanoparticle synthesis at 50, 60, and 70 °C. pNIPAM NPs synthesized at 60 °C presented an average hydrodynamic diameter similar to those developed at 70 °C, as previously established;^21,24^ core size was ∼180 nm (PDI = 0.06–0.31) and an increase was observed after shell synthesis (∼230 nm, PDI = 0.19–0.28) (Table 1 and Figure S2). At 50 °C, core–shell pNIPAM NP was not obtained, as no increase in size was observed after shell synthesis. Thus, 60 °C was selected as the synthesis temperature of the lipid-core–pNIPAM-shell hybrid nanoparticles to guarantee the maintenance of the characteristics of the lipid core and the shell polymerization; the same temperature was used for the control pNIPAM NP.

**Table 1:** Characterization of pNIPAM and Hybrid Nanoparticles Obtained at Different Temperatures

	formulation	temperature of synthesis (°C)	size	PDI	zeta potential (mV)	shell thickness (nm)
pNIPAM NP	core	70	169.8 ± 7.4	0.06 ± 0.01	–20.5 ± 2.0	47.3 ± 10.7
	core + shell		217.1 ± 5.6	0.28 ± 0.01	–26.5 ± 0.7	
	core	60	189.5 ± 6.0	0.31 ± 0.01	–16.2 ± 2.3	37.9 ± 10.8
	core + shell		227.4 ± 7.2	0.19 ± 0.02	–17.8 ± 1.4	
	core	50	221.9 ± 7.5	0.57 ± 0.02	–24.4 ± 0.6	a
	core + shell		181.7 ± 4.6	0.64 ± 0.05	–25.9 ± 1.8	
hybrid NP	core (NLC)	60	233.4 ± 7.2	0.18 ± 0.03	–11.9 ± 0.5	140.7 ± 9.7
	core (NLC) + shell		380.4 ± 4.4	0.28 ± 0.05	–15.7 ± 2.0	

a Not applicable.

Hybrid nanoparticles were formed through a two-step method: assembly of the lipid nanoparticle core followed by the synthesis of a pNIPAM shell around the preformed cores. The average diameter of the hybrid NP synthesized at 60 °C increased by ∼140 nm after addition of the pNIPAM shell, suggesting the formation of a lipid-core–polymeric-shell hybrid nanoparticle (Table 1 and Figure S2).

**Figure 1:**
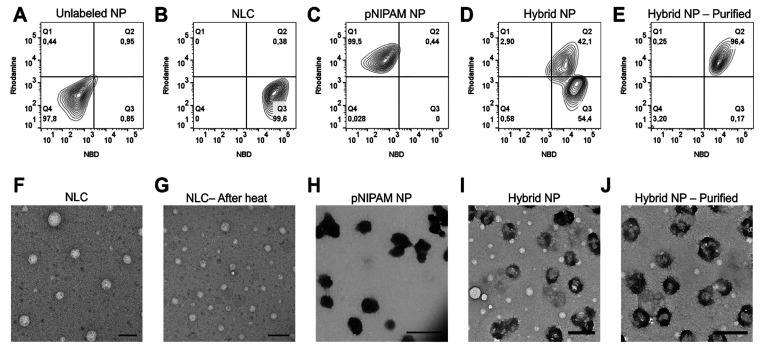
Confirmation of the hybrid core–shell nanoparticles. (A–E) Flow cytometry data of the various nanoparticles. Flow cytometry confirms that rhodamine labeled shells polymerize around NBD-labeled lipid cores forming a hybrid core–shell nanoparticle, rather than two distinct particles. (A) Unlabeled particles, (B) NBD-labeled nanostructured lipid carriers, (C) rhodamine-labeled pNIPAM particles, (D) hybrid nanoparticles, and (E) purified hybrid nanoparticles. (F–J) Transmission electron microscope images of (F) NLCs, (G) heated NLCs, (H) pNIPAM NPs, (I) hybrid nanoparticles, and (J) purified hybrid nanoparticles. Scale bars = 500 nm.

Additional verification of successful encapsulation of the NLCs, employed as the core, by the pNIPAM shells was provided by flow cytometry and transmission electron microscopy (TEM). Rhodamine B-labeled shells were polymerized around NBD-labeled lipid cores and colocalization of rhodamine and NBD was observed by flow cytometry (Figure 1A–E). Gates on the flow cytometer were created for nonlabeled nanoparticles and for those labeled with rhodamine, NBD, and both. Nanoparticles labeled with both fluorophores denote those in which the lipid core containing NBD was efficiently covered with the pNIPAM shell (which was labeled with rhodamine).

As can be observed in Figure 1A, particles with no fluorescent label were indicated in the lower left panel, while the lower right panel indicates the presence of particles labeled with NBD, indicative of the presence of the lipid core (Figure 1B). In the upper left panel, particles are labeled with rhodamine, which confirms the synthesis of the pNIPAM polymeric shell (Figure 1C). Finally, the top right panel indicates the presence of both markers, suggesting polymerization of the pNIPAM shell around the lipid core creating a hybrid nanoparticle (Figure 1D,E). However, as can be observed in panel D, immediately after the synthesis of hybrid nanoparticles, two populations were present, suggesting that a fraction of the lipid nanoparticles was not encapsulated by the polymeric shell (54.4%, count 54414). Core–shell hybrid nanoparticles accounted for 42.1% of the structures (count 42136). Purification of the nanoparticles via centrifugation resulted in a relatively pure population of core–shell nanoparticles (96.4%, count 95636; Figure 1E) and only small fractions of NBD-only (lipid core; 0.19% and count 191) or rhodamine B-only (new pNIPAM particles; 0.24% and count 242) particles.

Transmission electron microscopy images (Figure 1F–J) of all samples demonstrated approximately spherical nanoparticles with diameters similar to those reported by DLS; a slight size reduction of heated lipid nanoparticles (Figure 1G) and purification of hybrid nanoparticles by centrifugation (Figure 1J) were also further confirmed.

All particles displayed negative zeta potentials due to the incorporation of the sulfated AMPS in core–shell particles and the choice of lipids and surfactants in lipid nanoparticles (Table 1).^30,31^ Particle physicochemical properties did not vary pronouncedly upon drug incorporation or peptide attachment (Table S1). Additionally, the thermal responsiveness of pNIPAM and hybrid nanoparticles was maintained in drug-loaded and unloaded formulations (Figure S3). Swelling of the nanoparticles was observed; below the LCST and above the LCST the nanoparticles were readily and completely dispersed in water-based medium (PBS and water).

### Drug Encapsulation

The NLCs presented the highest paclitaxel encapsulation efficiency (EE%) of 92.9 ± 0.3%. After 4 h at 60 °C (simulating the conditions necessary for polymerization and production of hybrid NPs), the EE% of the NLC was reduced to 75.8 ± 1.0%, which might be attributed to drug diffusion with increasing temperature and its consequent premature release from the system.^32^ Similarly, paclitaxel encapsulation efficiency in hybrid nanoparticles was 76.6 ± 0.9%, suggesting that further drug loss did not occur beyond that related to the heating of the particles (after purification, the encapsulation efficiency was 68.3 ± 1.3%). pNIPAM nanoparticles displayed the lowest encapsulation efficiency (66.2 ± 0.2%). The reduced EE% can be attributed to the absence of the lipid core that most likely facilitates paclitaxel solubilization, incorporation, and retention inside the particle matrix.^33^ This represents one advantage of the hybrid nanoparticles over the pNIPAM NPs.

### Paclitaxel Release

Due to the importance of polymer degradation to drug release, rhodamine-labeled nanoparticles (pNIPAM and hybrid) were synthesized, and their degradation was assessed at pH 3.5 and 7.4 (Figure S4). We acknowledge that weak acidic conditions (pH 5.5–6.5) would be better to mimic the tumor microenvironment,^34^ but due to the importance of polymer degradation to drug release and to accelerate the process, pH 3.5 was selected to ensure nanoparticle degradation. As can be observed in Figure S4, following 48 h of incubation in PBS at pH 7.4, hybrid nanoparticles did not show significant signs of degradation; however, when exposed to pH 3.5, they began to degrade. One of the possible mechanisms associated with pH-mediated degradation is the cleavage of the disulfide bond within the cross-links, which we expect to occur due to the pNIPAM BAC crosslinker.^35,36^ The *N*,*N*′-bis(acryloyl cystamine) disulfide bond stability is greatly reduced in acidic medium, resulting in nanoparticle degradation and release of the entrapped compounds. Minimal absorbance was observed after 5 days of incubation at pH 3.5, suggesting complete degradation; at physiological pH, the degradation was approximately 75–80%. In previous work, we treated BAC-cross-linked pNIPAM nanoparticles with dithiothreitol (DTT) or acidic conditions.^21,37^ As expected, both DTT and acidic conditions resulted in particle dissolution when disulfide cross-links were present. However, when particles were cross-linked with *N*-methyl(bis-acrylamide) there was no degradation under similar conditions. In addition, we previously showed that the BAC-cross-linked pNIPAM nanoparticles degrade in endosomes over 8 days, demonstrating that the nanoparticles do degrade slowly under slightly acidic pH (∼5.5).^21^

**Figure 2:**
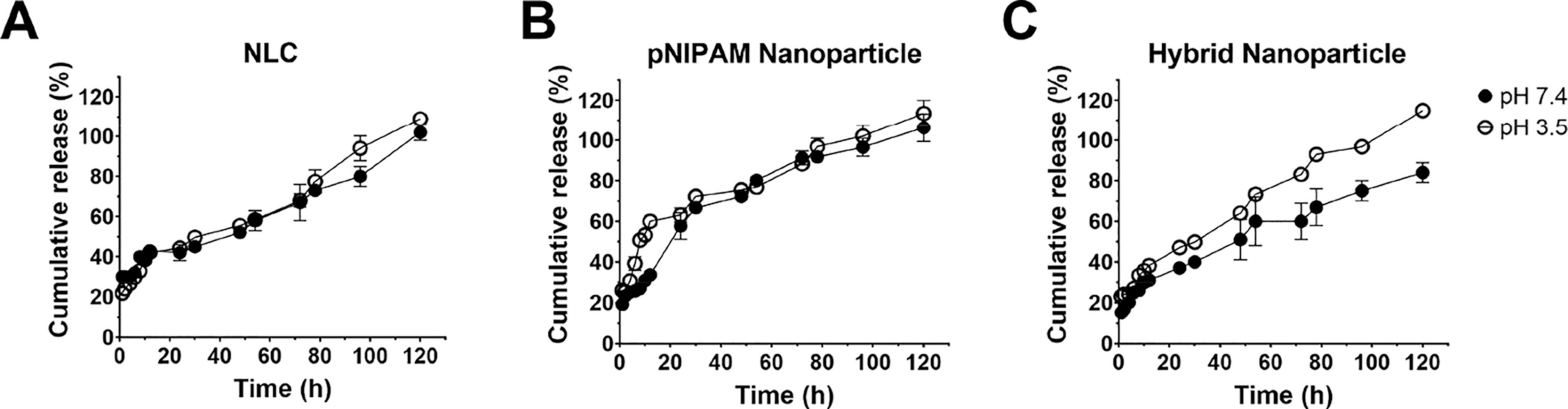
Paclitaxel release from (A) NLCs, (B) pNIPAM nanoparticles, and (C) hybrid nanoparticles in PBS, pH 3.5 and pH 7.4, over 5 days at 37 °C. Data shown as average ± standard deviation (*n* = 12).

Following degradation studies, paclitaxel release from the nanoparticles was assessed. As expected, the cumulative percentage of drug release was significantly higher at acidic pH (pH 3.5) compared to physiological pH (pH 7.4) for both hybrid and pNIPAM nanoparticles, especially within the first 24 h, while drug release from the NLC was not affected by pH changes (Figure 2). Paclitaxel sustained released was observed from all delivery systems. As shown in Figure 2B, the release rate of paclitaxel from pNIPAM nanoparticles was the highest with 57.7–63.1% of the drug released by 24 h and complete drug release over 78 h. The best fit for the release profile of paclitaxel from pNIPAM nanoparticles at physiological pH was obtained with the first-order or Higuchi models (*R*^2^ = 0.9854).^38^ Paclitaxel was released from the NLC at a lower rate compared to polymeric nanoparticles with ≈40% of the drug released by 24 h and ≈75% released over 78 h (Figure 2A). Compared to lipid and pNIPAM nanoparticles, hybrid systems exhibited the lowest release rate where only 37.2 ± 2.6% of paclitaxel was released over 24 h and over 84.0 ± 4.6% was released over 5 days at physiological pH (Figure 2C). For both lipid and hybrid nanoparticles, drug release was best described by the Higuchi model (*R*^2^ = 0.9735 and 0.9706, respectively), which is in accordance with previous studies. These results demonstrated that paclitaxel retention in hybrid nanoparticles was the highest, which may be attributed to the core–shell structure of the system and the multiple barriers that prevent premature drug release.^39^

### SILY Attachment to the Nanoparticles

**Figure 3:**
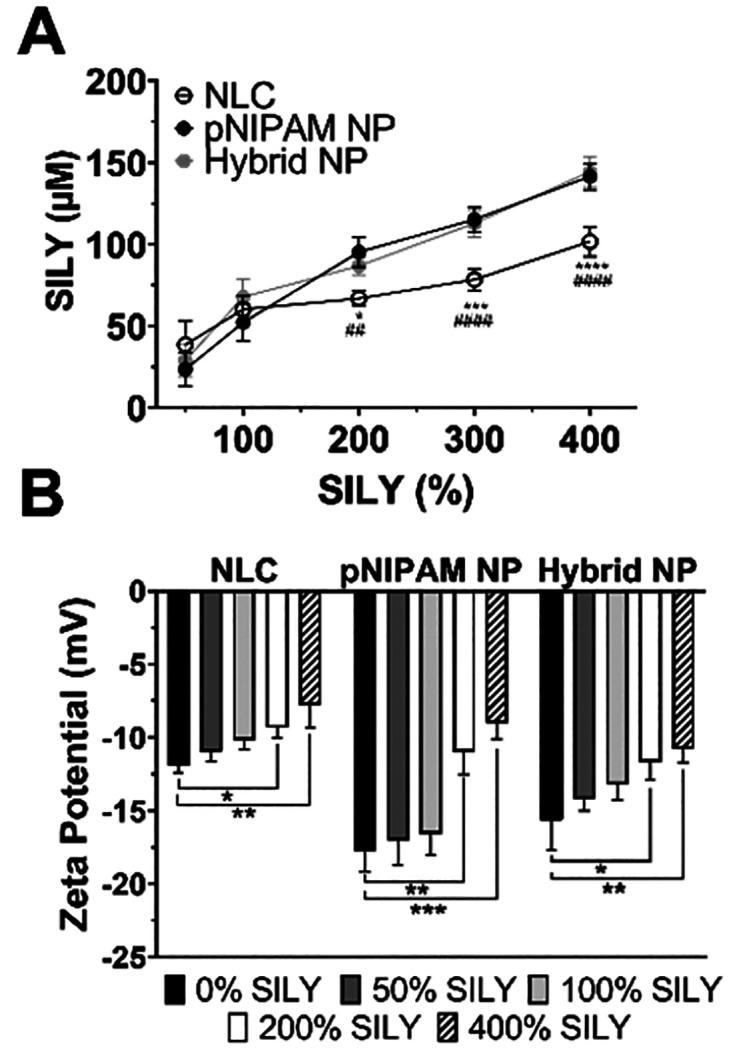
Nanoparticle functionalization with SILY. (A) SILY-binding to NLC, pNIPAM NPs, and hybrid NPs (*p < 0.05, ***p < 0.001, and ****p < 0.0001 compared to hybrid NPs; ^##^*p* < 0.01, and ^####^*p* < 0.0001 compared to pNIPAM NPs); (B) Zeta potential of particles modified with different ratios of SILY (*p < 0.05, **p < 0.01, and ***p < 0.001 compared to NP SILY 0%). Data show mean ± standard deviation 4–6 replicates.

At lower SILY to nanoparticle ratios (SILY 50 and 100% NPs), lipid nanoparticles presented the highest SILY coupling efficiency (Figure 3A).

As the ligand ratio increased, the conjugation efficiency decreased for the NLCs compared to hybrid and pNIPAM NPs. This can be attributed both to the steric hindrance effect on the lipid nanoparticle’s surface at higher ligand concentration^40^ and to the reduced number of carboxylic groups available for conjugation in the NLCs, which was limited to the behenic acids of Compritol 888.^41^ Nanoparticles with the pNIPAM shell were copolymerized with acrylic acid which provides more peptide attachment points resulting in a higher density of peptides on the surface.^25^

### SILY-NP Binding to Collagen

**Figure 4:**
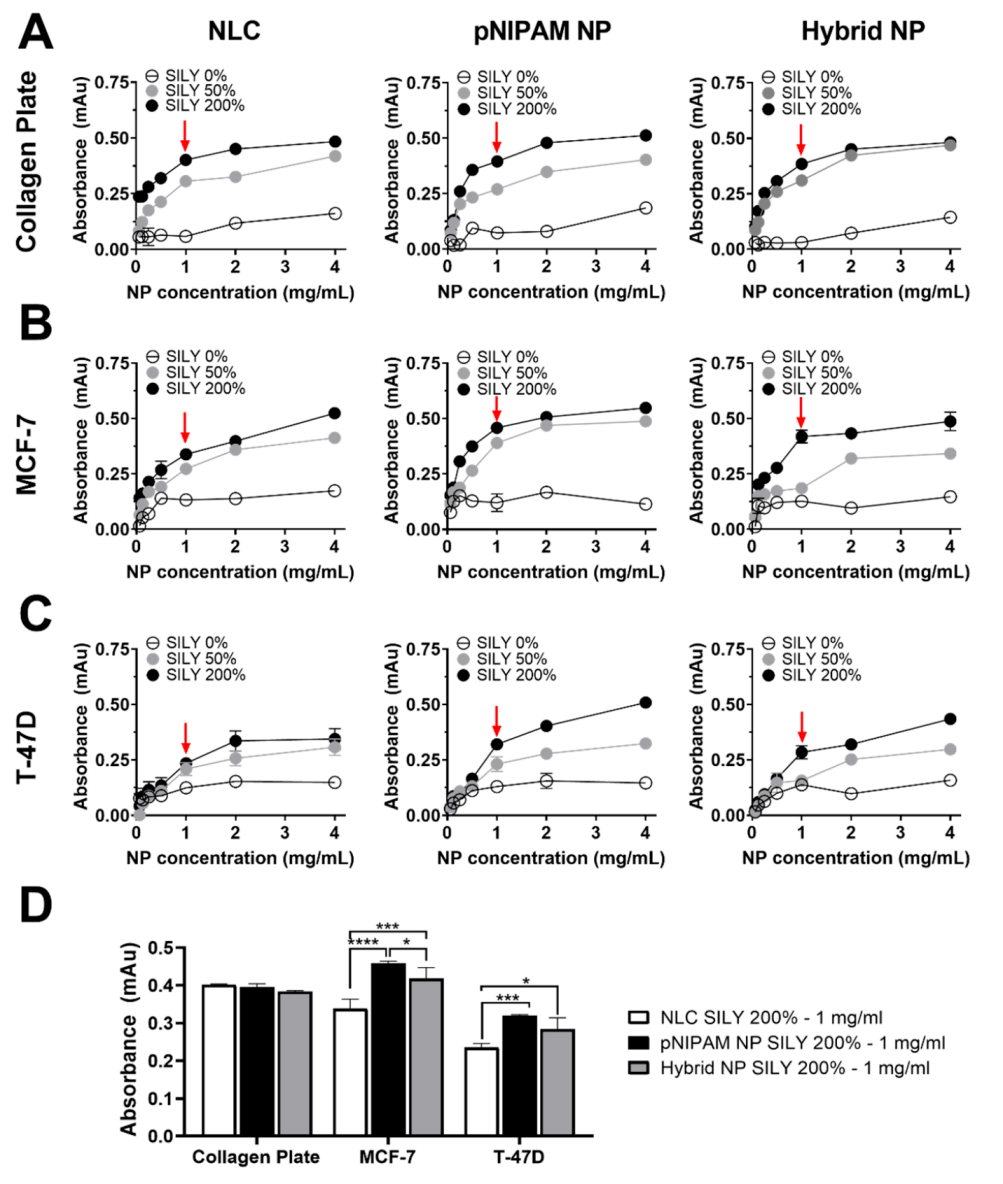
Collagen binding assay demonstrating the ability of SILY-modified nanoparticles to bind to collagen. (A, B, C) Nanoparticle ability to bind to collagen I-coated surfaces, naturally elaborated collagen from MCF-7 cells and T-47D cells, respectively (red arrows indicate the selected concentrations). (D) Ability of selected nanoparticles (SILY 200%) and concentration (1 mg/mL, in PBS) to bind to collagen. Particle binding increased with increases in conjugated SILY and NP concentration. NP SILY 0% did not show the ability to bind to the collagen plate.

To understand whether collagen binding increased with an increase in SILY surface functionalization, two collagen-binding studies were conducted. In the first study, the binding of SILY-modified nanoparticles to collagen was assessed in collagen-coated plates using a streptavidin–HRP colorimetric assay. For this experiment serial dilutions of NLCs, pNIPAM NPs, and hybrid NPs modified with 0% (control, nonmodified), 50%, and 200% SILY were employed. All SILY-modified particles bound to collagen, while nonmodified nanoparticles presented minimal attachment on the type I collagen surface as indicated by low absorbance values (Figure 4A). Higher SILY/nanoparticle ratios resulted in a correspondingly higher binding to collagen. Also, increasing the concentration of the formulation (NLCs, pNIPAM NPs, or hybrid NPs) resulted in a higher attachment. However, for most groups, a plateau for collagen binding was observed at approximately 1 mg/mL and higher formulation concentrations (2–4 mg/mL) did not provide advantages in terms of collagen binding. Among the different types of nanoparticles modified with the same amount of SILY, lipid nanocarriers present the lowest attachment to collagen surfaces. These results can be attributed to the slightly lower coupling efficiency of the SILY peptide on the surface of lipid nanoparticles, suggesting the advantage of building the pNIPAM shell to potentially improve targeting, selectivity, and local retention.

To evaluate the binding of SILY-NPs to collagen that was naturally produced by breast cancer cells, we first determined collagen secretion over time. As can be observed in Figure S5, collagen production increased from 1 to 5 days; no difference was observed comparing days 5 and 7. Additionally, collagen production by MCF-7 was higher at 1–4 days but became similar to that by T-47D at 5 and 7 days. Thus, cells were cultured for 5 days to maximize collagen production and ensure a similar level of production in both cell lines.

As demonstrated in Figure 4B,C, the results of nanoparticle binding to collagen synthesized by cells were similar to those obtained with the collagen-coated surfaces: hybrid and pNIPAM nanoparticles presented greater binding, which was mainly evident at the highest concentrations studied. Interestingly, SILY-modified nanoparticles showed a greater ability to attach to MCF-7 plates than T-47D; even though at day 5 both cell lines presented similar collagen expression levels. Considering these results, nanoparticles modified with 200% SILY at 1 mg/mL (Figure 4D) were selected for further experiments to assess the association of nanocarriers with cells.

**Figure 5:**
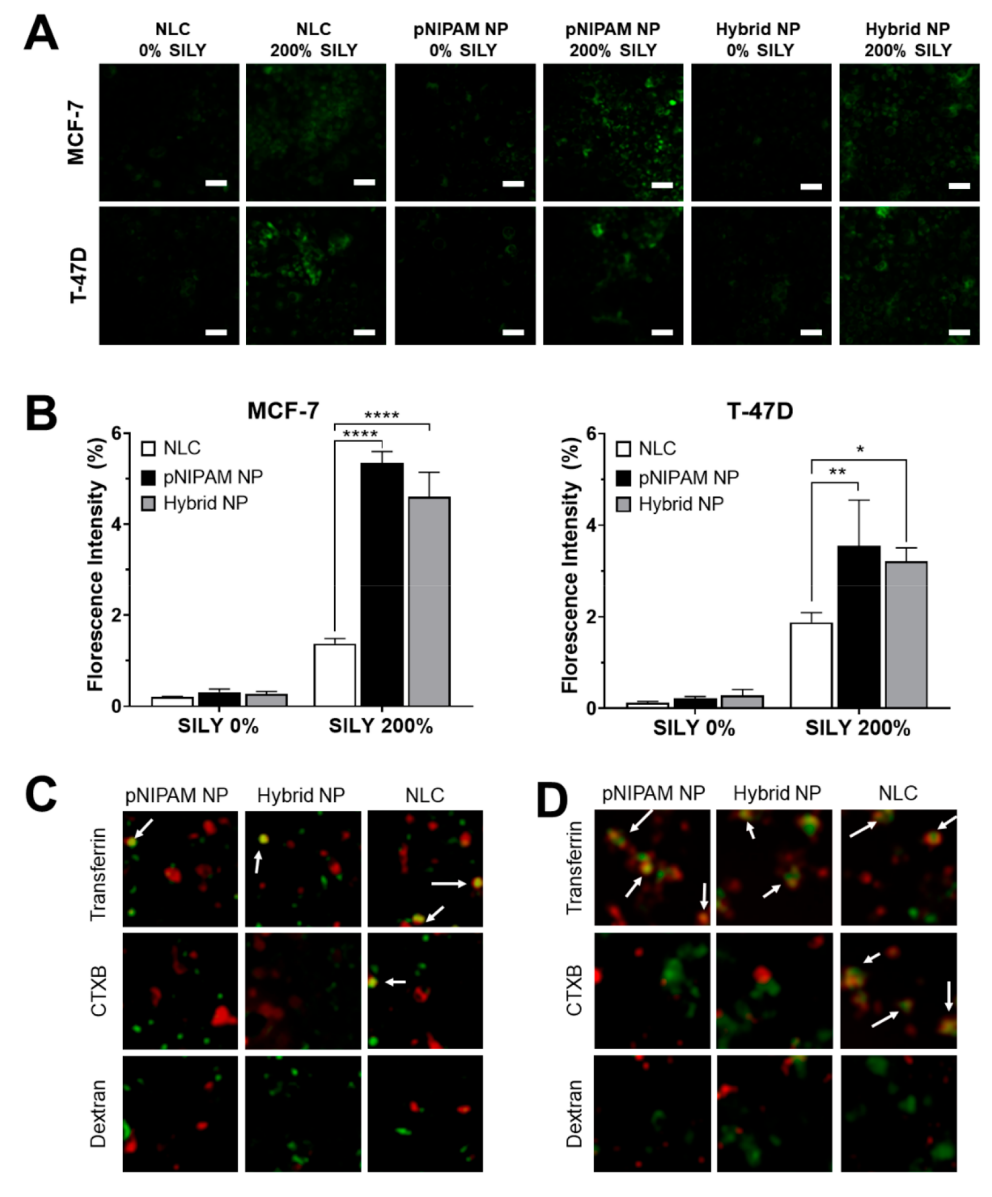
SILY-modified nanoparticles binding to naturally produced collagen and mechanism of endocytic uptake by MCF-7 and T-47D cells. Binding to cellular collagen was determined by FITC fluorescence quantification, and the endocytic uptake mechanism was determined by color overlap (yellow; denoted by white arrows) of FITC-labeled nanoparticles (green) and the markers of endocytosis (red). (A) Binding of fluorescent nanoparticles modified with SILY to collagen; (B) semiquantitative image analysis of the fluorescent area of nanoparticle binding (*p < 0.05, **p < 0.01, and ****p < 0.0001 compared to SILY-modified nanoparticles at the same treatment concentration); (C) endocytic uptake by MCF-7 cells; (D) endocytic uptake by T-47D cells. Scale bars = 50 μm.

Fluorescently labeled nanoparticle association with breast cancer cell cultures is shown in Figure 5A and confirms that SILY-modified nanocarriers successfully bind to naturally produced collagen, resulting in their association with cells. The type of association, whether adsorption, attachment, or internalization, was not further investigated. Fluorescence for pNIPAM and hybrid nanoparticles functionalized with SILY was similar and significantly higher than fluorescence for lipid nanoparticles in MCF-7 cells, indicating increased binding of nanoparticles containing the peptide attached to the pNIPAM shell, likely due to larger peptide density on the pNIPAM nanoparticle shell surface. Similar results were observed in T-47D cells. In both cell lines, fluorescence intensity was minimal in the absence of SILY (Figure 5B) confirming that the collagen-binding peptide is necessary for collagen attachment.

Thus, after verifying the ability of the nanoparticles to bind to naturally elaborated collagen, lipid, pNIPAM, and hybrid nanoparticles modified with 200% SILY were selected as optimized formulations and will be referred to as SILY-NLCs, SILY-pNIPAM NPs, and SILY-hybrid NPs, respectively.

### Characterization of Endocytic Uptake

To understand the relationship between the type of nanocarrier and mechanism of internalization, the colocalization of FITC-labeled nanoparticles (modified with SILY 200% at 1 mg/mL, in PBS) and markers of endocytosis were investigated. The internalization of the nanoparticles appeared to follow the same mechanism regardless of the studied cell line (Figure 5C,D). SILY-hybrid NPs and SILY-pNIPAM NPs showed localization with transferrin, the marker of clathrin-mediated endocytosis, in both MCF-7 and T-47D cells. Negligible colocalization was observed for the nanoparticles and cholera toxin subunit B (CTXB) or dextran, which are markers of caveolin-mediated endocytosis and macropinocytosis. In turn, NLCs colocalized with transferrin and CTXB, which suggest that are taken up by both clathrin- and caveolin-mediated endocytosis.

### Cytotoxicity Evaluation of Nanoparticles

In this experiment, cells were treated with various concentrations (0.003–50 mg/mL) of NLCs, pNIPAM NPs, and hybrid NPs modified with SILY, containing or not containing paclitaxel. Only SILY-modified nanoparticles were selected for this assay, since SILY surface functionalization and binding to collagen can influence several biological functions such as cell viability, survival, and proliferation, and not just promote active targeting of the system.^29^

#### Evaluation of Cell Viability in Monolayers (2D Model)

**Figure 6:**
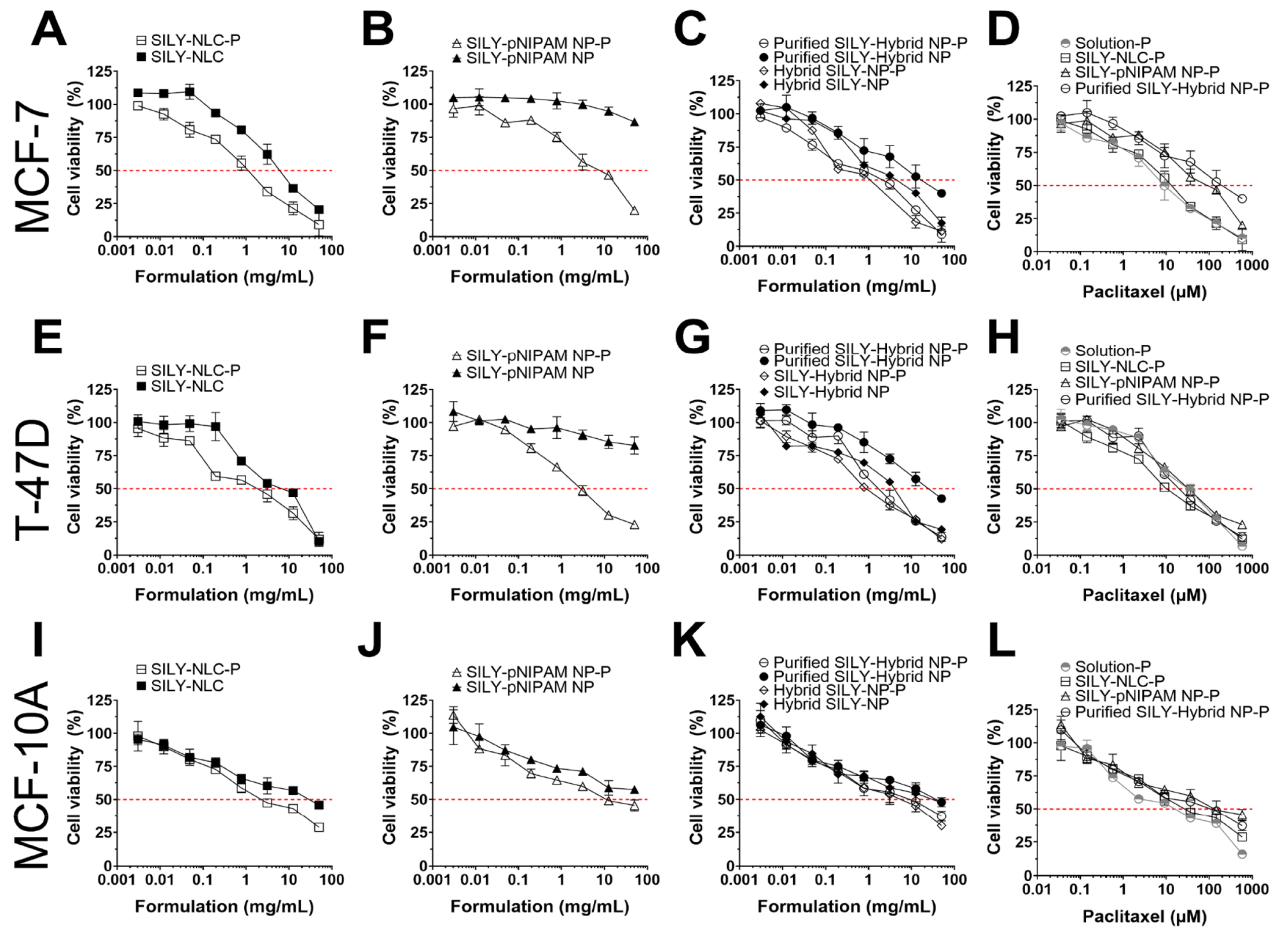
Viability of breast tumoral and nontumoral cells in monolayers after exposure to unloaded and paclitaxel-loaded nanoparticles solubilized in PBS. Cell viability was evaluated using the MTS assay after treatment for 72 h. (A–D) Comparison of the treatment of MCF-7 cells with (A) NLCs, (B) pNIPAM NPs, (C) hybrid NPs before and after purification, and (D) paclitaxel-loaded NPs and a drug solution; (E–H) Comparison of the treatment of T-47D cells with (E) NLCs, (F) pNIPAM NPs, (G) hybrid NPs before and after purification, and (H) paclitaxel-loaded NPs and a drug solution; (I–L) Comparison of the treatment of MCF-10A cells with (I) NLCs, (J) pNIPAM NPs, (K) hybrid NPs before and after purification, and (L) paclitaxel-loaded NPs and a drug solution. Data shown as the average ± standard deviation of 9–12 replicates in 3–4 independent experiments.

The cytotoxicity of drug-loaded and unloaded nanoparticles was tested between 0.003 and 50 mg/mL in MCF-7 and T-47D breast cancer cells (Figure 6). NLCs showed the greatest ability to reduce cell viability in both cell lines studied, with IC_50_ (half maximal inhibitory concentration) values of 1.1–1.4 mg/mL for drug-loaded and 6.6–7.9 mg/mL for unloaded particles (Figure 6A,E and Table 2). Interestingly, purification of the hybrid nanoparticles increased the formulation IC_50_ by up to 6.4-fold, which might be attributed to the removal of the free NLCs (Figure 6C,G). As observed in Figure 6B,F, unloaded pNIPAM nanoparticles did not reduce cell viability below 50% in any of the cells studied in the range of concentrations studied.

**Table 2:** Values of IC_50_ and Respective Confidence Intervals (95%, CI_95_) in MCF-7 and T-47D Cell Lines Cultured in Monolayer (2D Model) or Spheroids (3D Model)^a^

	IC_50_ in mg/mL of formulation (CI_95_ in mg/mL of formulation)					IC_50_ in μM of paclitaxel (CI_95_ in μM of paclitaxel)				
	MCF-7		T-47D		MCF-10A	MCF-7		T-47D		MCF-10A
formulation	2D	3D	2D	3D	2D	2D	3D	2D	3D	2D
hybrid NP	5 (2.3–6.4)	30.2 (28.4–60.5)	5.9 (2.8–6.5)	60.3 (48.3–95.3)	67.4 (61.7–76.5)	b	b	b	b	b
hybrid NP with paclitaxel	1.1 (0.4–2.7)	4.5 (2.8–10.5)	1.2 (0.8–1.6)	7.7 (3.7–14.4)	6.6 (5.9–10.2)	12.4 (10.0–22.7)	52.1 (33.0–75.7)	16.0 (13.2–18.6)	90.0 (69.3–140.5)	80.7 (54.6–108.8)
purified hybrid NP	18.7 (10.2–28.1)	132.0 (97.7–153.8)	37.8 (21.7–42.5)	142.1 (100.8–250.0)	76.4 (66.2–84.3)	b	b	b	b	b
purified hybrid NP with paclitaxel	2.1 (0.7–2.2)	7.7 (5.9–10.1)	3.1 (2.1–3.6)	9.9 (6.2–16.0)	6.4 (4.9–16.3)	14.2 (13.0–24.6)	90.6 (57.9–102.3)	24.6 (16.3–27.6)	116.5 (78.2–187.4)	104.9 (92.6–124.8)
pNIPAM NP						b	b	b	b	b
pNIPAM NP with paclitaxel	7.8 (6.0–9.8)	11.4 (8.5–19.4)	3.2 (2.3–4.7)	17.9 (10.0–32.9)	12.1 (10.6–20.2)	70.9 (55.0–95.4)	133.1 (83.2–145.0)	33.2 (26.9–46.0)	209.8 (147.4–305.4)	142.0 (131.5–187.0)
NLC	6.6 (4.5–9.1)	41.2 (34.6–62.4)	7.9 (4.7–9.9)	72.7 (58.2–101.0)	41.9 (31.1–54.3)	b	b	b	b	b
NLC with paclitaxel	1.1 (0.8–1.3)	5.0 (3.4–7.5)	1.4 (0.7–2.6)	6.9 (3.9–12.1)	3.3 (2.0–4.7)	11.2 (9.9–15.5)	59.0 (39.9–80.1)	14.6 (9.8–18.9)	80.3 (55.2–105.0)	39.1 (33.9–42.4)
solution paclitaxel	0.8 (0.7–1.0)	6.9 (5.2–11.2)	2.4 (1.8–3.4)	7.4 (5.4–16.9)	3.4 (3.0–5.3)	9.9 (7.8–12.5)	80.6 (59.6–101.3)	28.8 (20.6–40.4)	86.8 (59.9–103.5)	26.0 (16.3–43.9)

a The half maximal inhibitory concentration (IC_50_) was calculated using the MTS assay results after incubation with several treatments for 72 h.

b Not applicable.

As expected, paclitaxel incorporation in optimized nanoparticles increased formulation cytotoxicity up to 12.2-fold compared to that of unloaded nanoparticles; the most pronounced effect was observed for purified hybrid nanoparticles in T-47D cells (IC_50_ values of 3.1 and 37.8 mg/mL for paclitaxel-loaded and unloaded particles). Compared to a drug solution (Figure 6D,H), paclitaxel nanoencapsulation did not increase drug cytotoxicity, except for hybrid NPs and NLCs in T-47D cells (1.7–2.0-fold increase).

To study selectivity toward cancer cells, nontumoral mammary cells (MCF-10A) were also treated with optimized formulations and a drug solution (Figure 6I–L). Higher IC_50_ values were observed for NLCs (3.0-fold), pNIPAM NPs (3.8-fold), and hybrid nanoparticles (3.1–6-fold) in MCF-10A cells compared to tumor (MCF-7 and T-47D) cells, suggesting some selectivity in monolayers (Table 2).

#### Evaluation of Cell Viability in Spheroids (3D Model)

**Figure 7:**
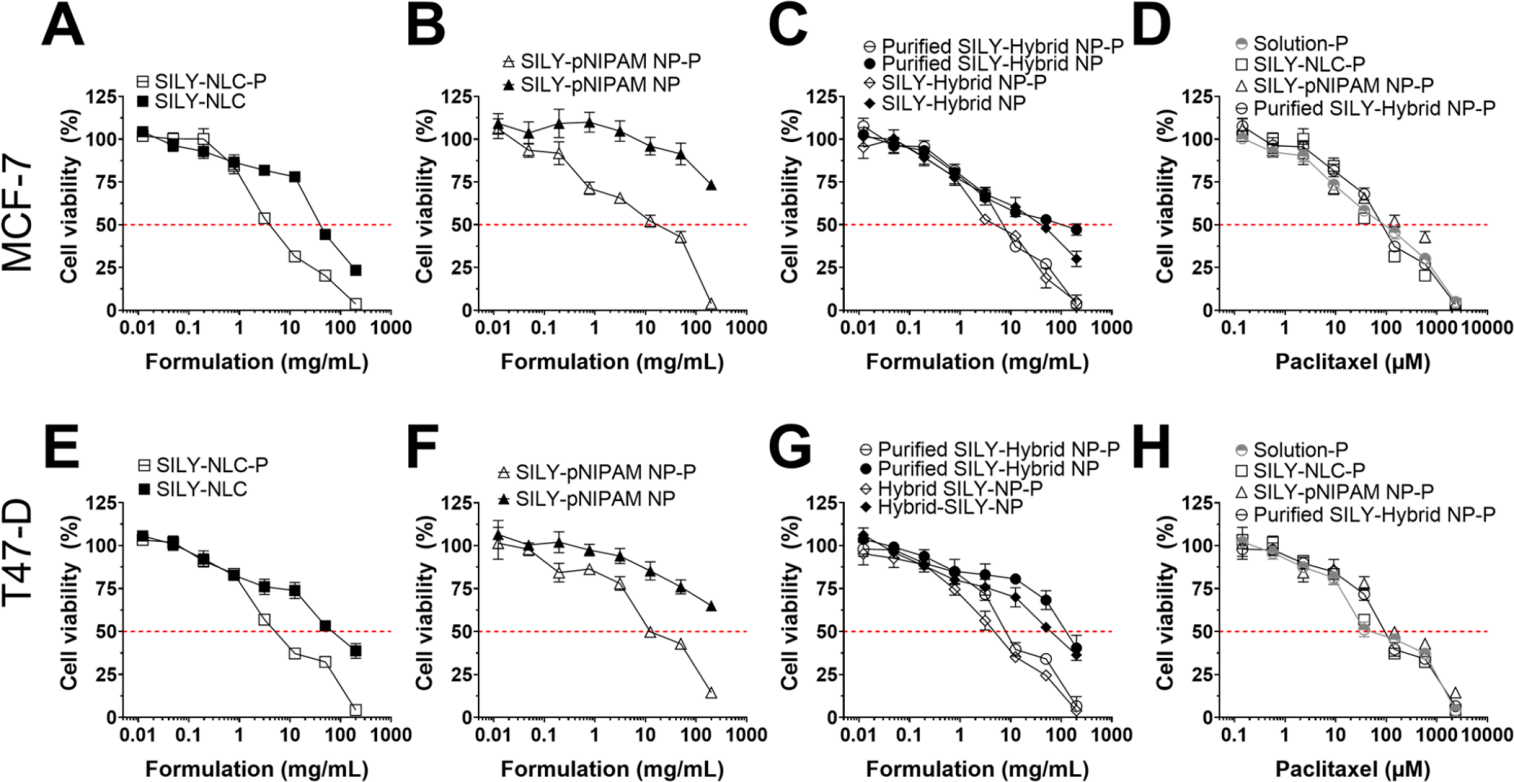
Viability of breast cancer spheroids after exposure to unloaded and paclitaxel-loaded nanoparticles solubilized in PBS. Cell viability was evaluated using the CellTiter-Glo 3D Cell Viability assay after treatment for 72 h. (A–D) Comparison of the treatment of MCF-7 cells with (A) NLCs, (B) pNIPAM nanoparticles, (C) hybrid nanoparticles before and after purification, and (D) paclitaxel-loaded nanoparticles or a drug solution; (E–H) Comparison of the treatment of T-47D cells with (E) NLCs, (F) pNIPAM nanoparticles, (G) hybrid nanoparticles before and after purification, and (H) paclitaxel-loaded nanoparticles or a drug solution. Data shown as the average ± standard deviation of 9–12 replicates in 3–4 independent experiments.

The cytotoxicity of the nanoparticles was also evaluated in three-dimensional models, which better represent the *in vivo* tumor microenvironment.^42^ As expected, the formulation IC_50_ values were higher than those obtained in cell monolayers (up to 10.2-fold), which can be attributed to diffusional barriers and limitations in drug penetration (Figure 7 and Table 2). As observed in cell monolayers, lipid and hybrid nanoparticles (before purification) showed the greatest ability to reduce cell viability in both cell lines (IC_50_ values of 5.0 and 4.5 mg/mL, respectively). Furthermore, paclitaxel encapsulation also increased the cytotoxicity of nanoparticles in spheroids from 6.0- to 17.1-fold; the most pronounced effect was observed for the purified hybrid nanoparticles in MCF-7 spheroids. Compared to the drug in solution, lipid and hybrid nanoparticles were able to reduce the formulation IC_50_ by up to 1.5-fold.

#### Evaluation of Cell Viability in the Transwell Model

Next, the cytotoxic effects of paclitaxel-loaded nanoparticles modified or not with SILY peptide were investigated in a co-culture model in which breast cancer cells had been cultured for 5 days to increase collagen production. This experiment was conducted to assess whether SILY-modified NPs, due to their ability to bind to collagen, had a lower impact on the viability of cells grown on the plate compared to cells grown on the transwell (which were in direct contact with the nanocarriers).

**Figure 8:**
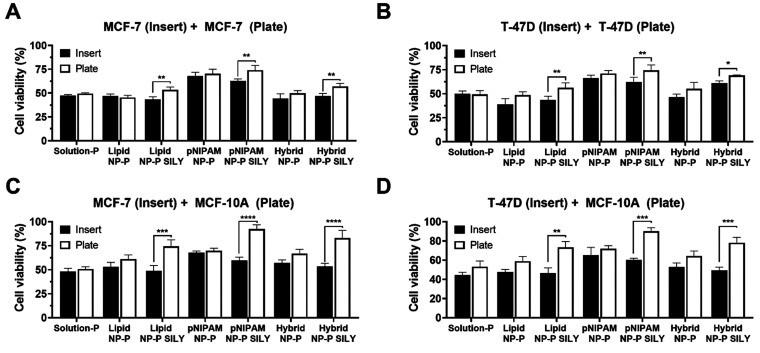
Cell viability assessed on MCF-7, T-47D, and MCF-10A cells with verious co-culture combinations after 72 h exposure to paclitaxel-loaded nanoparticles modified or not with SILY; paclitaxel solution was used as control. (A, B) Results of MCF-7 or T-47D cell lines cultured in the insert (black bars) and in the plate (white bars). (C, D) Depict results of MCF-10A co-cultured with MCF-7 and T-47D cells, respectively. Data shown as the average ± standard deviation of 3–6 replicates in 2–3 independent experiments.

This assessment was first conducted in the system in which tumor cells (MCF-7 or T-47D) were cultured on both the insert and 24-well plate. This enabled us to assess whether the cells in direct contact with the nanocarrier (in the transwell) were more susceptible to its effects. For both MCF-7 and T-47D cell lines, treatment with the paclitaxel solution or nonfunctionalized nanocarriers or paclitaxel solution also resulted in similar cell viability in both compartments. On the other hand, treatment with SILY-modified nanocarriers resulted in greater viability of the cells on the plate, even greater than in the experiment where tumor cells were cultured on the plate. SILY modified NLCs, pNIPAM NPs, and hybrid nanoparticles resulted in 1.52-, 1.55-, and 1.56-fold higher viability of MCF-10A cells, respectively, compared to cells treated with particles without SILY (Figure 8C). In co-culture with T-47D, viability enhancement of healthy cells was 1.57-, 1.50-, and 1.59-fold, for lipid, pNIPAM, and hybrid particles (Figure 8D). Thus, the greater viability of nontumor than tumor cells cultured on the plate suggest that collagen binding might improve selectivity toward cancer cells.

## Discussion

Nanotechnology provides great potential in the treatment of cancer, with several promising nanoparticle formulations being developed each year. In breast cancer therapy, nanotechnology-based formulations are already available in the clinic, including Doxil and Abraxane.^43^ However, applications of nanomedicine still face many challenges, including side effects due to systemic distribution and inefficient access of drugs to tumor sites. To address these shortcomings, several research groups have studied intraductal drug administration to enable the delivery of drugs through the ductal tree in a minimally invasive manner, while reducing drug levels systemically.^4,6^ However, medicine lacks formulations specially designed to maximize the benefits of this local route of administration. In this study, we developed a new generation of collagen-binding hybrid nanoparticles, aiming at local delivery, sustained release, and active targeting of breast tumoral tissue.

Hybrid nanoparticles with a polymeric core and a lipid shell have been described in the literature as promising drug delivery systems for anticancer therapy.^44^ In turn, hybrid nanoparticles that combine the advantages of an oil core with a polymeric shell remain poorly studied despite the clear advantages of this type of system. The lipid core allows drug solubilization and encapsulation, while the polymeric shell results in flexibility in terms of surface functionalization and responsiveness to physiological stimuli such as temperature or pH.^4,16−18^ In fact, the hybrid nanoparticles developed in this work presented superior encapsulation efficiency of paclitaxel than that of pNIPAM-only nanoparticles. Hybrid nanoparticles also presented higher surface modification with the SILY peptide compared to lipid-only nanoparticles. At the SILY ratio chosen (NP SILY 200%), building the pNIPAM shell around the lipid core increased the SILY conjugation by 1.3-fold.

The hybrid nanoparticles were approximately 1.6- and 1.2-fold larger in diameter than their polymeric or lipid-only counterparts, respectively, which may contribute to ductal retention.^14^ Although there is no consensus on the ideal size of nanoparticles for the intraductal administration of drugs, the previous literature suggests that, while respecting a range that does not obstruct the mammary ducts, larger particles have a longer local retention time, which can contribute to a reduction in the frequency of administration.^7,14^

Intraductal administration has been associated with increased mammary retention, which, in turn, leads to a considerable reduction in the dose of the drug that reaches systemic circulation and toxicity related to chemotherapy drugs.^13,45^ Thus, we do not anticipate that the nanoparticles would come into contact with blood and did not assess blood compatibility. Nevertheless, in previous studies, we demonstrated that pNIPAM nanoparticles do not generate hemolysis or alter blood coagulation, suggesting their hemocompatibility.^25,31^ Therefore, if part of the treatment undergoes systemic absorption, we still expect the nanoparticles to be safe.

Nanoparticles obtained with the pNIPAM polymeric shell maintained the ability to undergo phase transition around physiological temperature even when polymerized around the oily core. The pNIPAM shell undergoes hydrophobic collapse at body temperature thereby creating a porous diffusive barrier that contributes to the controlled release of the encapsulated drug.^46,47^ Furthermore, the acidic pH of tumors may aid in the release of compounds encapsulated in these nanoparticles, in preference to release in normal tissue.^48^ The pNIPAM shell has been copolymerized with the cross-linker agent *N*,*N*′-bis(acryloyl cystamine), and the disulfide bonds contained in this cross-linker are susceptible to cleavage at acidic pH, resulting in nanoparticle degradation and release of the encapsulated drug.^22^ In this study, we observed preferential degradation and release of paclitaxel when hybrid and pNIPAM nanoparticles were incubated at pH 3.5, when compared to physiological pH, which may contribute to increased drug concentration in the tumor tissue. Importantly, in previous studies the stability of peptide-loaded pNIPAM nanoparticles has also been demonstrated in a biological environment for up to a week, and during this period the polymeric shell is slowly degraded.^21^ While one notices that the nanoparticles do degrade under physiological conditions and release the encapsulated drug, nanoparticles can also be taken up by endocytosis. The uptake of the nanoparticles further facilitates local delivery of a high drug concentration to the cells. While lipid nanoparticles were internalized by clathrin- and caveolin-mediated endocytosis, hybrid and polymeric nanoparticles exhibit only clathrin-mediated endocytosis. Therefore, the synthesis of the polymeric shell and consequent change on nanoparticle surface seems to prevent caveolin-mediated endocytosis.^49^

Collagen-binding materials have wide clinical applications, including the treatment of breast cancer. Replacement of the normal extracellular matrix (ECM) by a tumor matrix is an essential part of tumorigenesis in breast cancer.^50^ Among the ECM changes, type I fibrillar collagen accumulation contributes to the formation of disorganized and highly proliferative cell clusters and is associated with an increased risk of cancer recurrence after ductal carcinoma *in situ* and poor response to therapy.^50,51^ Thus, we modified nanoparticles with a peptide that binds to type I collagen (SILY), aiming to obtain a targeted-therapy strategy. SILY-modified nanoparticles demonstrated the ability to bind to collagen-coated surfaces as well as collagen secreted by breast cancer cells. The pNIPAM shell synthesis increased the functionalization with SILY and the binding rate with collagen compared with those of lipid nanoparticles.

Interestingly, MCF-7 cell binding was superior to that to T-47D cells, even though on day 5 we observed similar collagen secretion. Although both cell lines have similar receptor expression (estrogen receptor and progesterone receptor positive), which could lead to similar collagen secretion patterns,^52^ some studies suggest that particularities in terms of expression of the gene that encodes collagen type 1 (COL1A1) may result in different densities of secreted collagen in cell culture.^53^ Thus, one hypothesis is that the collagen secreted by the MCF-7 cell may have a more mature and dense structure than that of T-47D cells, favoring the interaction with SILY.

Cell culture studies helped us to understand the cytotoxicity of the developed nanoparticles. We evaluated nanoparticle cytotoxicity in cell monolayers and spheroids, the latter of which is a model that better mimics tumor microenvironment *in vivo.*(42,54,55) Lipid nanoparticles showed the highest cytotoxicity, which may be related to the presence and availability of tributyrin from the lipid matrix. Salata et al. previously demonstrated an increase in the cytotoxicity of formulations in the presence of tributyrin in breast cancer cells, especially in paclitaxel-containing particles.^9^ Furthermore, it is important to mention that the cryoprotectant required for the lyophilization of lipid nanoparticles can influence the uptake of the particles. Coating nanoparticles with trehalose has already been shown to increase cellular internalization of nanoparticles, especially in tumor cells that overexpress the GLUT-1 receptor, which may result in increased cytotoxicity in such cells.^56^ Nanoparticles with the pNIPAM shell tolerate lyophilization without the need for a cryoprotectant, which reduces interferents in the cytotoxicity assay.^19^

Greater cytotoxicity was also observed for nonpurified hybrid nanoparticles. Considering that cytotoxicity was reduced after purification of hybrid nanoparticles, it is reasonable to suggest that the presence of lipid nanoparticles that were not encapsulated in the polymeric shell increased the cytotoxicity of the formulation, probably due to the greater availability of tributyrin. Cooperstein and colleagues previously demonstrated that pNIPAM-coated surfaces present high biocompatibility and are not cytotoxic to different cell types, which may further justify the cytotoxicity reduction in purified hybrid core–shell particles and the reduced cytotoxicity of unloaded pNIPAM particles.^57^ Finally, hybrid and polymeric nanoparticles without paclitaxel presented less cytotoxicity than the nanostructured lipid carrier, proving to be more suitable for applications in healthy women at high risk of developing breast cancer or in patients with pretumor lesions, that may also benefit from the intraductal route of administration.

Finally, new methods of co-culture of breast tumor cells with adipocytes or tumor-associated macrophages (TAMs) have been recently developed and presented in the literature, as a step forward in mimicking *in vivo* conditions.^58−60^ For example, the study by Wang and colleagues demonstrated that the viability of breast tumor cells (MDA-MB-231 and T41) was highly reduced after treatment with an anti-mammary hyperplasia drug, when grown in co-cultures with TAMs.^59^ In this study, we developed a noncontact co-culture system involving tumor and/or nontumoral breast cell lines, in which a permeable membrane allowed free exchange of media and soluble molecules. To the best of our knowledge, the evaluation of cell viability using indirect co-cultures of mammary tumoral and healthy cells has not been previously reported. Our co-culture results demonstrated that modifying the surface of nanoparticles with the SILY peptide resulted in greater cytotoxicity in cells in contact with the treatment compared to distant cells; we also observed higher cytotoxicity in tumor cells over healthy cells. Considering collagen secretion by tumor cells, these results suggest that SILY-modified nanoparticles bind to collagen, which leads to a more pronounced local cytotoxic effect in tumoral cells when compared to nonlocal healthy cells. Nanoparticles without surface modification consistently induced greater cytotoxic effect in normal cells cultured below the inserts after 72 h of treatment, suggesting the relevance of targeting the tumoral cells as was achieved here with the SILY collagen-binding peptide.

## Conclusion

We have developed hybrid nanoparticles for potential paclitaxel intraductal administration and breast cancer therapy, which were based on the encapsulation of lipid cores in polymeric pNIPAM shells, and compared the hybrid system with lipid-only and polymeric-only nanoparticles. The hybrid nanoparticles were further modified with collagen-binding peptide SILY for active targeting of the mammary tumoral tissue. We have demonstrated that encapsulating the lipid core in the polymeric pNIPAM shell increased nanoparticle functionalization with SILY. The release of paclitaxel seemed to follow the Higuchi release model, and sustained release can contribute to the effectiveness of therapy and a lower frequency of administration. The evaluation of the nanoparticles in tumor and nontumor breast cells showed that the hybrid formulation exhibited higher cytotoxicity in tumor cells compared to a control pNIPAM nanoparticle, while increasing the viability in distant nontumoral cells compared to nanostructured lipid carriers. This study provides a promising novel system for ductal carcinoma *in situ* therapy, highlighting the possibility of a local and less invasive treatment.

## Experimental Section

### Materials

Hydrazide modified collagen-binding peptide RRANAALKAGELYKSILYGSG-hydrazide (SILY-hydrazide, molecular weight 2252.6 kDa, 80% purity) was purchased from Innopep (San Diego, CA, USA) and a biotin-labeled version of the peptide (SILY_biotin_, molecular weight 2422.9 kDa, 92.4% purity) was purchased from GenScript (Piscataway, NJ, USA). Paclitaxel was purchased from Cayman Chemical (Ann Arbor, MI, USA), and soy phosphatidylcholine (PC) and 1-palmitoyl-2-(6-((7-nitro-2-1,3-benzoxadiazol-4-yl)amino)hexanoyl)-*sn*-glycero-3-phosphocholine (NBD-PC) were purchased from Avanti Polar Lipids (Alabaster, AL, USA). Poly(*N*-Isopropylacrylamide) (≥98%, pNIPAM) and rhodamine B were acquired from Polysciences Inc. (Warrington, PA, USA). Polysorbate 80 (Tween 80), tributyrin, sodium dodecyl sulfate (SDS; 10% w/v in water), 2-acrylamido-2-methyl-1-propanesulfonic acid (99%, AMPS), fluorescein *o*-acrylate (98%, FITC), potassium persulfate (99%, KPS) and *N*,*N*-bis(acryloyl)cystamine (98%, BAC) were acquired from Sigma-Aldrich (St. Louis, MO, USA). Acrylic acid (AAc), 1-ethyl-3-(3-(dimethylamino)propyl)carbodiimide hydrochloride (EDC), and *N*-hydroxysuccinimide (NHS) were purchased from ThermoFisher Scientific (Waltham, MA, USA). Glyceryl behenate (Compritol 888 ATO) was kindly supplied by Gattefosse (Saint-Priest, France). pNIPAM and BAC were stored under nitrogen at 4 °C. All water used in synthesis, dialysis, and testing was treated by a Milli-Q system (Millipore, Billerica, MA, USA; 18.2 MΩ·cm resistivity).

### Methods

#### Nanoparticle Synthesis

In this study, the properties of the hybrid NPs in terms of drug release, collagen binding, cell uptake mechanisms, and cytotoxicity were compared to the NLCs and the polymeric (pNIPAM) nanoparticles. Thus, these three types of nanocarriers were produced and characterized, as described in the following sections.

##### Nanostructured Lipid Carrier Formation

NLCs were obtained by a fusion emulsification technique as previously optimized.^30^ The oil phase (10% of the NLC content) consisted of Compritol 888, tributyrin, and phosphatidylcholine (3.5:3.5:3 w/w/w). The aqueous phase (consisting of PBS and Tween 80, 3% w/w) was added to the melted oil phase under vortex mixing, and the final mixture was immersed in a water bath for temperature control and probe sonicated for 20 min (50 s on and 30 s off) using 40% amplitude (QSonica Q700, Newtown, CT, USA). Paclitaxel-loaded lipid nanoparticles (NLC-P) were obtained by dissolving the drug in the oil-phase before aqueous phase inclusion (final concentration of 1%, w/w of the formulation). All lipid nanoparticles were lyophilized using trehalose as cryoprotectant.^61,62^ A trehalose solution at 10% was added to the formulation (1:1, v/v), and the mixture was frozen at −20 °C for 12 h, followed by 24 h of lyophilization.

##### pNIPAM Nanoparticle Synthesis

pNIPAM (polymeric) nanoparticles (pNIPAM NPs) were obtained using a precipitation polymerization reaction.^21−24^ First, a range of temperatures (50, 60, or 70 °C) for NP synthesis was tested to ensure that polymerization would occur without changes to NLC characteristics due to lipid melting. A temperature of 70 °C was established as the upper limit since previous differential scanning calorimetry results revealed the melting of the lipid core at ∼70 °C.^30,41^ In turn, the lower limit was 50 °C, which is the temperature at which potassium persulfate (KPS) decomposes, initiating the polymerization of the pNIPAM-based nanoparticle;^63^ 60 °C represents an intermediate temperature.

To obtain the nanoparticles, a pNIPAM shell was built around a core of the polymer, followed by core removal by dialysis for subsequent loading of the drug into the shell. Briefly, 30 mL of Milli-Q water was heated at predetermined temperatures in a three-neck round-bottom flask under nitrogen for 20 min. To create the pNIPAM core, 394.7 mg of pNIPAM and 164 μL of SDS were dissolved in 5 mL of Milli-Q water and added to the flask. To initiate the reaction, 67.4 mg of potassium persulfate was dissolved in 2 mL of Milli-Q water and added to the flask. After reaction for 2 h, nanoparticle cores were obtained and exposed to atmospheric oxygen for 45 min to terminate free radicals. For pNIPAM shell synthesis, the reaction flask was placed under nitrogen again for 20 min. Next, 794.5 mg of pNIPAM, 75.6 mg of AMPS, 164 μL of SDS, and 4.81 μL of AAc were dissolved in 5 mL of Milli-Q water and added to the flask. Finally, 24.1 mg of BAC (a labile disulfide cross-linker) was dissolved in 10 mL of Milli-Q water and added to the flask, followed by 33.7 mg of KPS dissolved in 2 mL of Milli-Q water. Additional 5 mL aliquots of the “shell solution” were added 30, 60, and 90 min after the initial polymerization. After 4 h, the pNIPAM core–shell nanoparticles were cooled at room temperature. Then, the nanoparticles were dialyzed against Milli-Q water using a 15 kDa MWCO dialysis membrane (SpectraPor, Spectrum Laboratories, San Francisco, CA, USA) at 4 °C for 7 days for core removal and future drug loading. Nanoparticles consisting of a pNIPAM shell will be referred to as pNIPAM NPs. Finally, the dialyzed nanoparticles were lyophilized and stored at room temperature.

Paclitaxel-loaded pNIPAM nanoparticles (pNIPAM NP-P) were obtained by a swelling method.^22^ One milligram of the lyophilized pNIPAM NPs and 2 mg of paclitaxel were incubated in 1 mL of 100% ethanol at 4 °C, to ensure pNIPAM shell swelling and drug loading by diffusion, as previously described.^21^ Briefly, when placed at 4 °C, pNIPAM shell polymers become hydrophilic and expand, allowing drug loading into the particle. After 24 h, the particles were centrifuged for 1 h at 18,0000*g* and 25 °C. The pelleted nanoparticles were resuspended in 1 mL of Milli-Q water, frozen, and lyophilized; the supernatant was collected for paclitaxel encapsulation efficiency (EE%) determination.

##### Hybrid Nanoparticle Synthesis

Hybrid nanoparticles were prepared in two steps: fabrication of the nanostructured lipid carrier (NLC, as core), followed by the synthesis of the pNIPAM shell. Briefly, the NLCs were diluted at 1:10 (v/v) in Milli-Q water immediately after production. The diluted particles were added to a 3-neck round-bottom flask and heated at 60 °C under nitrogen for 20 min. To create the polymeric shell, pNIPAM, AMPS, SDS, AAc, BAC, and KPS (see the previous section for details) were added to the flask to initialize the polymerization. Additional aliquots of the “shell solution” were added 30, 60, and 90 min after the initial polymerization, and the reaction was paused after 4 h. The hybrid nanoparticles were cooled at room temperature overnight and then purified via centrifugation at 4000 rpm for 10 min to remove lipid cores that were not encapsulated in the pNIPAM shell. In all experiments, the purified hybrid nanoparticles were used with the exception of the cytotoxicity experiments, in which we compared the nanoparticles before and after purification. Next, the nanoparticles were frozen, lyophilized, and stored at room temperature. Nanoparticles obtained with lipid nanoparticles as core and pNIPAM shell will be referred to as hybrid NPs. For paclitaxel-loaded hybrid nanoparticles (hybrid NP-P), NLC-P was used as core.

To ensure that the lipid nanoparticles were stable at the temperature necessary for pNIPAM shell synthesis, their aqueous dispersion was incubated for 4 h at 50, 60, and 70 °C, and changes to their size were assessed using dynamic light scattering (see Supporting Information, S1).

#### Nanoparticle Characterization

Size distribution and ζ potential were determined using a Nano-ZS90 Zetasizer (Malvern, Westborough, MA). Nanoparticles were dissolved at 1 mg/mL in Milli-Q water in a disposable polystyrene cuvette and subjected to at least three individual measurements with 11 runs each. The nanoparticles were also subjected to temperature trends to evaluate their thermosensitive behavior. Size determination was performed from 17 °C to 41 °C in 2 °C increments with equilibration for 2 min between each step.

The structure and morphological aspects of the nanoparticles were assessed using transmission electron microscopy (TEM) at the UC Davis School of Medicine on an FEI CM120 (Hillsboro, OR). Discharged TEM grids were placed on a 7 μL droplet of nanoparticles resuspended in Milli-Q water for 10 min prior to staining with uranyl acetate (2%, v/v). Samples were dried and imaged at room temperature. Confirmation of core–shell structures was further assessed using fluorescently labeled nanoparticles and flow cytometry (Aurora, Cytek Biosciences, Fremont, CA). The lipid cores were labeled with NBD-PC (1% of the total PC concentration, Avanti Polar Lipids, Alabaster, AL, USA). To obtain fluorescently labeled pNIPAM shells (hybrid and pNIPAM NPs), 0.1 mol % rhodamine B isothiocyanate dissolved in 1 mL of DMSO was injected following pNIPAM, AMPS, BAC, AAc, and SDS addition and before shell polymerization initiation.^21^ All samples were kept in the dark for further experiments.

#### Drug Loading and Release

The efficiency of paclitaxel encapsulation in the nanoparticles was evaluated indirectly by centrifugation at 18,000*g* for 1 h.^21^ Supernatant was collected, and EE% was obtained by the following equation: EE% = (*C*_total_ – *C*_free_)/*C*_free_ × 100, where *C*_total_ is the initial amount of paclitaxel added to the nanoparticle and *C*_free_ is the amount of nonencapsulated paclitaxel detected in the supernatant.

Paclitaxel release was assessed using a Franz diffusion cell (PermeGear V6-CB, Hellertown, PA, USA) equipped with a Corio CD-BC4 heating circulator (Julabo, Sellback, Germany). The nanoparticles were placed in the donor compartment of the diffusion cell and separated from the receptor phase using a cellulose dialysis membrane (MWCO 15,000 Da, Spectrum Laboratories, San Francisco, CA, USA). Phosphate buffered saline (pH 7.4 or 3.5) containing 1% polysorbate 80 was selected as the receptor phase and maintained at 37 °C under stirring (200 rpm). A proper sink condition was maintained throughout the release studies, in which drug concentration in the receptor phase reached less than 27% of its solubility.^64^ Aliquots of the receptor phase (0.25 mL) were withdrawn at predetermined time points up to 120 h and paclitaxel was quantified using a SpectraMax M5 plate reader (Molecular Devices, Sunnyvale, CA) at 230 nm as previously described.^65,66^

Due to the importance of nanoparticle degradation for drug release from hybrid and pNIPAM NPs, rhodamine-labeled nanoparticles were synthesized to further understand their degradation following exposure to PBS pH 7.4 and 3.5.^22^ Briefly, unlabeled pNIPAM cores were obtained as previously described. Next, 1 mol % rhodamine B isothiocyanate was predissolved in 3% DMSO in Milli-Q water and then added to the shell solution and allowed to equilibrate for 30 min before the polymerization of the shell was initiated with the addition of KPS. Nanoparticles were dialyzed for core removal and lyophilized. For hybrid nanoparticles, unlabeled lipid nanoparticles were used as cores, and the same procedure was used for rhodamine-labeled pNIPAM shell synthesis. Then, nanoparticles were dissolved at the final concentration of 1 mg/mL in PBS and the absorbance was monitored over 7 days using a SpectraMax M5 (Molecular Devices, Sunnyvale, CA) at 544 nm.

#### Nanoparticle Functionalization with SILY

Collagen-binding nanoparticles were obtained using EDC/NHS activation chemistry.^23,67^ First, 10 mg of lyophilized nanoparticles (lipid, pNIPAM, or hybrid NPs) was activated for 30 min by dissolving at 5 mg/mL in a coupling buffer consisting of 0.1 M MES, 8 M urea, 10 mM EDC, and 20 mM NHS at pH 4.5. Then, different molecular equivalents of hydrazide SILY (SILY/AAc) were added to the solution and allowed to react for 90 min; considering that 10% of the carboxylic acids were potentially accessible within the nanoparticle, 0, 0.5, 1, 2, and 4 mol equiv of SILY were evaluated (named NP-SILY 0, 50, 100, 200, and 400%, respectively).

For subsequent collagen binding affinity assays, 1% of the total concentration of SILY was added as SILY_biotin_. The resulting SILY-conjugated nanoparticles were purified using tangential flow filtration (KrosFlo KR2i, Spectrum Laboratories, Dominguez, CA, USA) equipped with a 10 kDa filter.^21^ Following purification, nanoparticles were frozen, lyophilized, and stored at room temperature. Coupling efficiency of the peptide to the nanoparticles was confirmed by measuring the aromatic residues (Tyr) using a NanoDrop OneC UV–vis spectrophotometer (Thermo Fisher Scientific, MA, USA) at 280 nm. In addition, changes in the zeta potentials of the nanoparticles were investigated to better understand whether the binding of the positively charged peptide neutralizes part of the negative charge on the surface of the nanocarriers.

#### SILY-NP Binding to Collagen

The ability of SILY-modified nanoparticles to bind to collagen was assessed in two independent experiments. In the first experiment, collagen-coated 96-well plates were employed as substrate for SILY and biotinylated particles were used.^22,23,25^ In the second assay, nanoparticle ability to bind to collagen produced by cells was evaluated using breast cancer cell lines.^25^

For the first experiment, collagen-coated 96-well plates (Corning Biocoat, VWR, Radnor, PA, USA) were blocked with 1% bovine serum albumin (BSA), treated with SILY-NPs dissolved in 1% BSA in 1× PBS at different concentrations (0–4 mg/mL), and the plate was incubated on a plate shaker at 37 °C and 200 rpm for 30 min.^22,23,25^ After rinsing three times with 1× PBS, 100 μL of a diluted streptavidin–HRP solution (R&D Systems, MN, USA) was added, and the plate was incubated for 20 min on a plate shaker at 200 rpm and room temperature. The solution was removed, and the plate was rinsed three times with 1× PBS, followed by incubation for 20 min at 200 rpm with 100 μL of a reagent color solution (R&D Systems, MN, USA). Finally, 50 μL of 2 N H_2_SO_4_ was added to stop the reaction, followed by determination of the absorbances at 450 and 540 nm on a SpectraMax M5 plate reader.

To verify nanoparticle ability to bind to naturally produced collagen from breast cancer cells, MCF-7 and T-47D cells (see Cell Culture section) were treated with FITC-labeled nanoparticles (SILY 0% NPs and SILY 200% NPs, 1 mg/mL in PBS); to obtain FITC-labeled particles, 0.1 mol % FITC was added to the formulation, as described for the other fluorescent markers.^25^ To determine the cell incubation time necessary for collagen production, secreted collagen was assayed directly from confluent culture medium grown for 1–7 days, according to the manufacturer’s instructions (Soluble Collagen Quantification Assay Kit, Cat. No. CS0006). Based on the results, the cells were seeded at 5 × 10^4^ cells/cm^2^ on 24-well plates and incubated for 5 days for collagen secretion. Subsequently, the cells were treated with FITC-labeled SILY-NPs for 24 h, before rinsing 3 times with PBS to remove unbound nanoparticles. A Keyence BZ9000 (Itasca, IL, USA) microscope was used to acquire fluorescence images; 5–6 images of each treatment were evaluated using ImageJ to measure the fluorescence area and obtain semiquantitative measures of SILY-NP binding to collagen. In addition, SILY-NP binding to naturally produced collagen was quantified using biotinylated particles.^25^ MCF-7 and T-47D cells were seeded at 5 × 10^4^ cells/cm^2^ on 96-well plates (see Cell Culture section for details). After 5 days, cells were treated with serial dilutions of NP-SILY and incubated for 30 min. Quantitative assessment of collagen binding was performed using the streptavidin–HRP colorimetric assay, as studied on collagen-coated plates.^25^

#### Cell Culture

MCF-7 and T-47D (luminal A breast cancer cells) and MCF-10A (nontumor mammary epithelial cells) were used in this study. Breast cancer cells were cultured in DMEM-GlutaMAX (Gibco, Carlsbad, CA) medium supplemented with 10% fetal bovine serum (FBS, Gibco, Carlsbad, CA) and 1% penicillin and streptomycin (100 U/mL and 100 μg/mL Gibco, Carlsbad, CA). MCF-10A cells were cultured in DMEM-GlutaMAX (Gibco, Carlsbad, CA) medium supplemented with 5% horse serum (Gibco, Carlsbad, CA), 0.02 μg/mL Epidermal Growth Factor, 0.1 μg/mL cholera toxin, 10 μg/mL insulin, 0.5 μg/mL hydrocortisone, and 1% penicillin and streptomycin (100 U/mL and 100 μg/mL Gibco, Carlsbad, CA). Cells were grown at 37 °C in 5% CO_2_ atmosphere, and passage was performed at 80% confluence.

#### Characterization of Endocytic Uptake: Colocalization Study

To elucidate the type of endocytic uptake of the nanoparticles, markers of the three major types of endocytosis were used.^68^ Texas red labeled dextran (macropinocytosis), Alexa Fluor 594 labeled cholera toxin subunit B (caveolae-mediated endocytosis), and Texas red labeled transferrin (clathrin-mediated endocytosis) were used at final concentrations of 1 mg/mL, 10 μg/mL, and 25 μg/mL, respectively. T-47D and MCF-7 cells were treated with FITC-labeled NPs and with markers of endocytosis for 1 h. For all cellular experiments, nanoparticles were solubilized in PBS to control the osmotic balance.^69^ Next, the cells were washed three times with media and then incubated with 100 μL of trypan blue (0.4% solution) to cover the bottom of the well and quench extracellular FITC signal for 1 min. Cells were washed five times with media, followed by imaging using a Keyence BZ9000 (Itasca, IL, USA) microscope. Unlabeled nanoparticles were included for autofluorescence assessment.

#### Cytotoxicity Evaluation in Cell Monolayers (2D Model)

To assess cell viability after treatment with nanoparticles, the colorimetric MTS assay (CellTiter 96 AQ_ueous_ One Solution Cell Proliferation Assay, Promega) was used.^29,70^ The cells (MCF-7, T-47D, and MCF-10A) were seeded in 96-well plates at a concentration of 5 × 10^4^ cells/well in DMEM GlutaMAX culture medium and incubated at 37 °C in a 5% CO_2_ incubator for 24 h. Then, cells were treated with serial dilutions of unloaded or paclitaxel-loaded nanoparticles (0.003–50 mg/mL, in PBS). After 72 h, 20 μL of CellTiter 96 One Solution Reagent was added into each well containing 100 μL of culture medium. Cells were incubated for 3 h, followed by determination of absorbance at 490 nm on a SpectraMax M5 plate reader. Cell viability was expressed as percentage of live cells compared to control cells and GraphPad Prism 8 was used for estimation of drug concentrations necessary to reduce cell viability to 50% (IC_50_).

#### Cytotoxicity Evaluation in Spheroids (3D Model)

The liquid overlay technique was employed to obtain MCF-7 and T-47D spheroids.^8,9^ Prior to seeding of the cells at 5 × 10^3^ cells/well, the bottoms of 96-well plates were coated with 50 μL of an agarose solution (1%). The plates were centrifuged at 1000 rpm for 7 min and incubated at 37 °C for 5 days. Spheroid formation was confirmed using a Keyence BZ9000 (Itasca, IL, USA) microscope. The spheroids were treated with unloaded or paclitaxel-loaded nanoparticles at different concentrations for 72 h. Cell viability was determined by ATP quantification using the commercial CellTiter-Glo 3D Cell Viability kit (Promega, Madison, WI, USA) according to the manufacturer’s instructions.

#### Cytotoxicity Evaluation in a Co-culture Model

To evaluate the influence of SILY and collagen-binding ability of nanoparticles on cytotoxic effects in cells not directly exposed to treatment, a co-culture system using cell inserts was developed.^71,72^ We hypothesized that (i) this model might mimic how intraductal treatment would enable tumor cells in the ducts to be exposed to the drug-loaded nanocarrier more directly than healthy cells located more deeply in the tissue and (ii) the nanocarrier presented selectivity toward tumor cells. In this system, tumor cells were cultured on cell inserts that were placed in 24-well cell culture plates in which tumor or nontumor cells were seeded.

Tumoral (T-47D or MCF-7) cells were seeded into 24-well cell culture inserts on a semipermeable support membrane (cell culture insert, 0.4 μm pore size; Falcon, Corning, New-York, USA) at a density of 1.7 × 10^5^ cells/cm^2^ (i.e., 5 × 10^4^ cells/insert, corresponding to a confluent cell monolayer) and were incubated for 5 days under normal culture conditions. Then, the cell culture inserts were placed into 24-well plates containing the same breast cancer cell line or MCF-10A (nontumor) cells seeded 24 h before at a density of 3 × 10^5^ cells/mL (i.e., 4 × 10^5^ cells/well). Both inserts and wells were supplied with media; the final volume of the medium was 700 μL in the bottom of each well and 200 μL in the hanging inserts. Cells seeded on the top insert were treated with concentrations of the nanoparticles modified or not with SILY corresponding to the IC_50_ of the drug in solution (concentrations inhibiting 50% of cell viability) previously obtained on the cell viability assay with monolayers (Table 2). The cytotoxic effects were explored after 72 h of exposure to treatments using the colorimetric MTS assay.

#### Data Analysis

All data are expressed as mean ± standard deviation. Statistical analyses were carried out using computer software GraphPad Prism 8 (San Diego, CA, USA). The data were analyzed for differences using paired and unpaired *t* tests for comparisons between two groups. For multiple comparisons, ANOVA was employed with a Tukey post hoc test. Differences were considered significant when *p* < 0.05.

## Supporting Information Available

Lipid nanoparticle stability under heating, DLS intensity-based size distribution histograms of the nanoparticles, DLS hydrodynamic diameter temperature sweeps of lipid, polymeric, and hybrid nanoparticles, degradation of pNIPAM shell incubated in PBS, quantification of collagen production by breast cancer cells, and size, PDI, and zeta potential of drug-loaded, unloaded, and SILY-modified particles (PDF)

## Abbreviations

NLC: nanostructured lipid carrier
pNIPAM: poly(*N*-isopropylacrylamide)
DCIS: ductal carcinoma *in situ*
PC: soy phosphatidylcholine
SDS: sodium dodecyl sulfate
AMPS: 2-acrylamido-2-methyl-1-propanesulfonic acid
FITC: fluorescein *o*-acrylate
KPS: potassium persulfate
BAC: *N*,*N*-bis(acryloyl)cystamine
AAc: acrylic acid
NLC-P: paclitaxel-loaded nanostructured lipid carrier
pNIPAM NP: polymeric nanoparticle
pNIPAM NP-P: paclitaxel-loaded polymeric nanoparticle
hybrid NP: hybrid nanoparticle
hybrid NP-P: paclitaxel-loaded hybrid nanoparticle
SILY-NLC: lipid nanoparticle modified with 200% SILY
SILY-pNIPAM NP: polymeric nanoparticle modified with 200% SILY
SILY-hybrid NP: hybrid nanoparticle modified with 200% SILY
CTXB: cholera toxin subunit B
MES: 2-(*N*-morpholino)ethanesulfonic acid
HRP: horseradish peroxidase
NBD: nitrobenzodiazole

## References

[ref1] Arnold M.; Morgan E.; Rumgay H.; Mafra A.; Singh D.; Laversanne M.; Vignat J.; Gralow J. R.; Cardoso F.; Siesling S.; et al. Current and future burden of breast cancer: Global statistics for 2020 and 2040. Breast 2022, 66, 15–23. 10.1016/j.breast.2022.08.010. 36084384 PMC9465273

[ref2] Badve S. S.; Gökmen-Polar Y. Ductal carcinoma in situ of breast: update 2019. Pathology 2019, 51 (6), 563–569. 10.1016/j.pathol.2019.07.005. 31472981 PMC6788802

[ref3] Ward E. M.; DeSantis C. E.; Lin C. C.; Kramer J. L.; Jemal A.; Kohler B.; Brawley O. W.; Gansler T. Cancer statistics: Breast cancer in situ. CA Cancer J. Clin 2015, 65 (6), 481–495. 10.3322/caac.21321. 26431342

[ref4] Sapienza Passos J.; Dartora V. F. M. C.; Cassone Salata G.; Draszesski Malagó I.; Lopes L. B. Contributions of nanotechnology to the intraductal drug delivery for local treatment and prevention of breast cancer. Int. J. Pharm. 2023, 635, 122681 10.1016/j.ijpharm.2023.122681. 36738808

[ref5] Murata S.; Kominsky S. L.; Vali M.; Zhang Z.; Garrett-Mayer E.; Korz D.; Huso D.; Baker S. D.; Barber J.; Jaffee E.; et al. Ductal access for prevention and therapy of mammary tumors. Cancer Res. 2006, 66 (2), 638–645. 10.1158/0008-5472.CAN-05-4329. 16423990

[ref6] Kuang X. W.; Liu J. H.; Sun Z. H.; Sukumar S.; Sun S. R.; Chen C. Intraductal Therapy in Breast Cancer: Current Status and Future Prospective. J. Mammary Gland Biol. Neoplasia 2020, 25 (2), 133–143. 10.1007/s10911-020-09453-4. 32577880

[ref7] Gu Z.; Al-Zubaydi F.; Adler D.; Li S.; Johnson S.; Prasad P.; Holloway J.; Szekely Z.; Love S.; Gao D.; et al. Evaluation of intraductal delivery of poly(ethylene glycol)-doxorubicin conjugate nanocarriers for the treatment of ductal carcinoma in situ (DCIS)-like lesions in rats. Journal of Interdisciplinary Nanomedicine 2018, 3 (3), 146–159. 10.1002/jin2.51. 30443411 PMC6220801

[ref8] Dartora V. F. C.; Salata G. C.; Passos J. S.; Branco P. C.; Silveira E.; Steiner A. A.; Costa-Lotufo L. V.; Lopes L. B. Hyaluronic acid nanoemulsions improve piplartine cytotoxicity in 2D and 3D breast cancer models and reduce tumor development after intraductal administration. Int. J. Biol. Macromol. 2022, 219, 84 10.1016/j.ijbiomac.2022.07.162. 35907458

[ref9] Salata G. C.; Lopes L. B. Phosphatidylcholine-Based Nanoemulsions for Paclitaxel and a P-Glycoprotein Inhibitor Delivery and Breast Cancer Intraductal Treatment. Pharmaceuticals (Basel) 2022, 15 (9), 1110 10.3390/ph15091110. 36145331 PMC9503599

[ref10] Farante G.; Toesca A.; Magnoni F.; Lissidini G.; Vila J.; Mastropasqua M.; Viale G.; Penco S.; Cassano E.; Lazzeroni M.; et al. Advances and controversies in management of breast ductal carcinoma in situ (DCIS). Eur. J. Surg Oncol 2022, 48 (4), 736–741. 10.1016/j.ejso.2021.10.030. 34772587

[ref11] van Seijen M.; Lips E. H.; Thompson A. M.; Nik-Zainal S.; Futreal A.; Hwang E. S.; Verschuur E.; Lane J.; Jonkers J.; Rea D. W.; et al. Ductal carcinoma in situ: to treat or not to treat, that is the question. Br. J. Cancer 2019, 121 (4), 285–292. 10.1038/s41416-019-0478-6. 31285590 PMC6697179

[ref12] Rosenberg S. M.; Gierisch J. M.; Revette A. C.; Lowenstein C. L.; Frank E. S.; Collyar D. E.; Lynch T.; Thompson A. M.; Partridge A. H.; Hwang E. S. “Is it cancer or not?” A qualitative exploration of survivor concerns surrounding the diagnosis and treatment of ductal carcinoma in situ. Cancer 2022, 128 (8), 1676–1683. 10.1002/cncr.34126. 35191017 PMC9274613

[ref13] Stearns V.; Mori T.; Jacobs L. K.; Khouri N. F.; Gabrielson E.; Yoshida T.; Kominsky S. L.; Huso D. L.; Jeter S.; Powers P.; et al. Preclinical and clinical evaluation of intraductally administered agents in early breast cancer. Sci. Transl Med. 2011, 3 (106), 106ra108 10.1126/scitranslmed.3002368. PMC3616888 22030751

[ref14] Joseph M. K.; Islam M.; Reineke J.; Hildreth M.; Woyengo T.; Pillatzki A.; Baride A.; Perumal O. Intraductal Drug Delivery to the Breast: Effect of Particle Size and Formulation on Breast Duct and Lymph Node Retention. Mol. Pharmaceutics 2020, 17 (2), 441–452. 10.1021/acs.molpharmaceut.9b00879. 31886676

[ref15] Gu Z.; Gao D.; Al-Zubaydi F.; Li S.; Singh Y.; Rivera K.; Holloway J.; Szekely Z.; Love S.; Sinko P. J. The effect of size and polymer architecture of doxorubicin-poly(ethylene) glycol conjugate nanocarriers on breast duct retention, potency and toxicity. Eur. J. Pharm. Sci. 2018, 121, 118–125. 10.1016/j.ejps.2018.04.033. 29698706

[ref16] Bou S.; Wang X.; Anton N.; Bouchaala R.; Klymchenko A. S.; Collot M. Lipid-core/polymer-shell hybrid nanoparticles: synthesis and characterization by fluorescence labeling and electrophoresis. Soft Matter 2020, 16 (17), 4173–4181. 10.1039/D0SM00077A. 32286601

[ref17] Oh K. S.; Lee K. E.; Han S. S.; Cho S. H.; Kim D.; Yuk S. H. Formation of core/shell nanoparticles with a lipid core and their application as a drug delivery system. Biomacromolecules 2005, 6 (2), 1062–1067. 10.1021/bm049234r. 15762679

[ref18] Huynh N. T.; Passirani C.; Saulnier P.; Benoit J. P. Lipid nanocapsules: a new platform for nanomedicine. Int. J. Pharm. 2009, 379 (2), 201–209. 10.1016/j.ijpharm.2009.04.026. 19409468

[ref19] Kokardekar R. R.; Shah V. K.; Mody H. R. PNIPAM Poly (N-isopropylacrylamide): A Thermoresponsive “Smart” Polymer in Novel Drug Delivery Systems. Internet Journal of Medical Update 2012, 59–62.

[ref20] Gandhi A.; Paul A.; Sen S. O.; Sen K. K. Studies on thermoresponsive polymers: Phase behaviour, drug delivery and biomedical applications. Asian Journal of Pharmaceutical Sciences 2015, 10 (2), 99–107. 10.1016/j.ajps.2014.08.010.

[ref21] Deloney M.; Smart K.; Christiansen B. A.; Panitch A. Thermoresponsive, hollow, degradable core-shell nanoparticles for intra-articular delivery of anti-inflammatory peptide. J. Controlled Release 2020, 323, 47–58. 10.1016/j.jconrel.2020.04.007. PMC9930616 32278830

[ref22] McMasters J.; Poh S.; Lin J. B.; Panitch A. Delivery of anti-inflammatory peptides from hollow PEGylated poly(NIPAM) nanoparticles reduces inflammation in an ex vivo osteoarthritis model. J. Controlled Release 2017, 258, 161–170. 10.1016/j.jconrel.2017.05.008. PMC5535751 28495577

[ref23] McMasters J.; Panitch A. Prevention of Collagen-Induced Platelet Binding and Activation by Thermosensitive Nanoparticles. AAPS J. 2015, 17 (5), 1117–1125. 10.1208/s12248-015-9794-9. 26070443 PMC4540739

[ref24] Kosinski A. M.; Brugnano J. L.; Seal B. L.; Knight F. C.; Panitch A. Synthesis and characterization of a poly(lactic-co-glycolic acid) core + poly(N-isopropylacrylamide) shell nanoparticle system. Biomatter 2012, 2 (4), 195–201. 10.4161/biom.22494. 23507885 PMC3568105

[ref25] McMasters J.; Panitch A. Collagen-binding nanoparticles for extracellular anti-inflammatory peptide delivery decrease platelet activation, promote endothelial migration, and suppress inflammation. Acta Biomater 2017, 49, 78–88. 10.1016/j.actbio.2016.11.023. 27840254 PMC5253112

[ref26] Goldbloom-Helzner L.; Hao D.; Wang A. Developing Regenerative Treatments for Developmental Defects, Injuries, and Diseases Using Extracellular Matrix Collagen-Targeting Peptides. Int. J. Mol. Sci. 2019, 20 (17), 4072 10.3390/ijms20174072. 31438477 PMC6747276

[ref27] Bodelon C.; Mullooly M.; Pfeiffer R. M.; Fan S.; Abubakar M.; Lenz P.; Vacek P. M.; Weaver D. L.; Herschorn S. D.; Johnson J. M.; et al. Mammary collagen architecture and its association with mammographic density and lesion severity among women undergoing image-guided breast biopsy. Breast Cancer Res. 2021, 23 (1), 105 10.1186/s13058-021-01482-z. 34753492 PMC8579610

[ref28] Roată C.-E.; Iacob S̨.; Morăraşu S.; Livadaru C.; Tudorancea I.; Luncă S.; Dimofte M.-G. Collagen-Binding Nanoparticles: A Scoping Review of Methods and Outcomes. Crystals 2021, 11 (11), 1396 10.3390/cryst11111396.

[ref29] Hao D.; Lu L.; Song H.; Duan Y.; Chen J.; Carney R.; Li J. J.; Zhou P.; Nolta J.; Lam K. S.; et al. Engineered extracellular vesicles with high collagen-binding affinity present superior in situ retention and therapeutic efficacy in tissue repair. Theranostics 2022, 12 (13), 6021–6037. 10.7150/thno.70448. 35966577 PMC9373818

[ref30] Passos J. S.; Martino L. C.; Dartora V. F. C.; Araujo G. L. B.; Ishida K.; Lopes L. B. Development, skin targeting and antifungal efficacy of topical lipid nanoparticles containing itraconazole. Eur. J. Pharm. Sci. 2020, 149, 105296 10.1016/j.ejps.2020.105296. 32151706

[ref31] Bartlett R. L.; Medow M. R.; Panitch A.; Seal B. L. Hemocompatible Poly(NIPAm-MBA-AMPS) Colloidal Nanoparticles as Carriers of Anti-inflammatory Cell Penetrating Peptides. Biomacromolecules 2012, 13 (4), 1204–1211. 10.1021/bm300173x. 22452800

[ref32] Rehman M.; Ihsan A.; Madni A.; Bajwa S. Z.; Shi D.; Webster T. J.; Khan W. S. Solid lipid nanoparticles for thermoresponsive targeting: evidence from spectrophotometry, electrochemical, and cytotoxicity studies. Int. J. Nanomedicine 2017, 12, 8325–8336. 10.2147/IJN.S147506. 29200845 PMC5701611

[ref33] Ma P.; Mumper R. J. Paclitaxel Nano-Delivery Systems: A Comprehensive Review. Journal of Nanomedicine and Nanotechnology 2013, 4 (2), 1000164 10.4172/2157-7439.1000164. 24163786 PMC3806207

[ref34] Chiche J.; Brahimi-Horn M. C.; Pouyssegur J. Tumour hypoxia induces a metabolic shift causing acidosis: a common feature in cancer.. J. Cell Mol. Med. 2010, 14 (4), 771–794. 10.1111/j.1582-4934.2009.00994.x. 20015196 PMC3823111

[ref35] Gyarmati B.; Némethy Á.; Szilágyi A. Reversible disulphide formation in polymer networks: A versatile functional group from synthesis to applications. Eur. Polym. J. 2013, 49 (6), 1268–1286. 10.1016/j.eurpolymj.2013.03.001.

[ref36] Ahiabu A.; Serpe M. J. Rapidly Responding pH- and Temperature-Responsive Poly(N-Isopropylacrylamide)-Based Microgels and Assemblies. ACS Omega 2017, 2 (5), 1769–1777. 10.1021/acsomega.7b00103. 31457540 PMC6640923

[ref37] Poh S.; Lin J. B.; Panitch A. Release of anti-inflammatory peptides from thermosensitive nanoparticles with degradable cross-links suppresses pro-inflammatory cytokine production. Biomacromolecules 2015, 16 (4), 1191–1200. 10.1021/bm501849p. 25728363 PMC4839979

[ref38] Son G.; Lee B.; Cho C. Mechanisms of drug release from advanced drug formulations such as polymeric-based drug-delivery systems and lipid nanoparticles. Journal of Pharmaceutical Investigation 2017, 47, 287–296. 10.1007/s40005-017-0320-1.

[ref39] Sivadasan D.; Sultan M. H.; Madkhali O.; Almoshari Y.; Thangavel N. Polymeric Lipid Hybrid Nanoparticles (PLNs) as Emerging Drug Delivery Platform-A Comprehensive Review of Their Properties, Preparation Methods, and Therapeutic Applications. Pharmaceutics 2021, 13 (8), 1291 10.3390/pharmaceutics13081291. 34452251 PMC8399620

[ref40] Du J.; Li L. Which one performs better for targeted lung cancer combination therapy: pre- or post-bombesin-decorated nanostructured lipid carriers?. Drug Deliv 2016, 23 (5), 1799–1809. 10.3109/10717544.2015.1099058. 26455787

[ref41] Aburahma M. H.; Badr-Eldin S. M. Compritol 888 ATO: a multifunctional lipid excipient in drug delivery systems and nanopharmaceuticals. Expert Opin Drug Deliv 2014, 11 (12), 1865–1883. 10.1517/17425247.2014.935335. 25152197

[ref42] Imamura Y.; Mukohara T.; Shimono Y.; Funakoshi Y.; Chayahara N.; Toyoda M.; Kiyota N.; Takao S.; Kono S.; Nakatsura T.; et al. Comparison of 2D- and 3D-culture models as drug-testing platforms in breast cancer. Oncol. Rep. 2015, 33 (4), 1837–1843. 10.3892/or.2015.3767. 25634491

[ref43] Wu D.; Si M.; Xue H. Y.; Wong H. L. Nanomedicine applications in the treatment of breast cancer: current state of the art. Int. J. Nanomedicine 2017, 12, 5879–5892. 10.2147/IJN.S123437. 28860754 PMC5566389

[ref44] Mohanty A.; Uthaman S.; Park I.-K. Utilization of Polymer-Lipid Hybrid Nanoparticles for Targeted Anti-Cancer Therapy. Molecules 2020, 25 (19), 4377 10.3390/molecules25194377. 32977707 PMC7582728

[ref45] Zhang B.; Love S. M.; Chen G.; Wang J.; Gao J.; Xu X.; Wang Z.; Wang X. The safety parameters of the study on intraductal cytotoxic agent delivery to the breast before mastectomy. Chin J. Cancer Res. 2014, 26 (5), 579–587. 10.3978/j.issn.1000-9604.2014.10.06. 25400424 PMC4220263

[ref46] Liu R.; Fraylich M.; Saunders B. R. Thermoresponsive copolymers: from fundamental studies to applications. Colloid and Polymer Science 2009, 287, 627–643. 10.1007/s00396-009-2028-x.

[ref47] Mi P. Stimuli-responsive nanocarriers for drug delivery, tumor imaging, therapy and theranostics. Theranostics 2020, 10 (10), 4557–4588. 10.7150/thno.38069. 32292515 PMC7150471

[ref48] Chen M.; Chen C.; Shen Z.; Zhang X.; Chen Y.; Lin F.; Ma X.; Zhuang C.; Mao Y.; Gan H.; et al. Extracellular pH is a biomarker enabling detection of breast cancer and liver cancer using CEST MRI. Oncotarget 2017, 8 (28), 45759–45767. 10.18632/oncotarget.17404. 28501855 PMC5542224

[ref49] Wang J.; Ayano E.; Maitani Y.; Kanazawa H. Tunable Surface Properties of Temperature-Responsive Polymer-Modified Liposomes Induce Faster Cellular Uptake. ACS Omega 2017, 2 (1), 316–325. 10.1021/acsomega.6b00342. 31457232 PMC6640984

[ref50] Lepucki A.; Orlinska K.; Mielczarek-Palacz A.; Kabut J.; Olczyk P.; Komosinska-Vassev K. The Role of Extracellular Matrix Proteins in Breast Cancer. J. Clin Med. 2022, 11 (5), 1250 10.3390/jcm11051250. 35268340 PMC8911242

[ref51] Egeblad M.; Rasch M. G.; Weaver V. M. Dynamic interplay between the collagen scaffold and tumor evolution. Curr. Opin Cell Biol. 2010, 22 (5), 697–706. 10.1016/j.ceb.2010.08.015. 20822891 PMC2948601

[ref52] Mah E. J.; Lefebvre A.; McGahey G. E.; Yee A. F.; Digman M. A. Collagen density modulates triple-negative breast cancer cell metabolism through adhesion-mediated contractility. Sci. Rep 2018, 8 (1), 17094 10.1038/s41598-018-35381-9. 30459440 PMC6244401

[ref53] Meng C.; He Y.; Wei Z.; Lu Y.; Du F.; Ou G.; Wang N.; Luo X. G.; Ma W.; Zhang T. C.; et al. MRTF-A mediates the activation of COL1A1 expression stimulated by multiple signaling pathways in human breast cancer cells. Biomed Pharmacother 2018, 104, 718–728. 10.1016/j.biopha.2018.05.092. 29807221

[ref54] Muguruma M.; Teraoka S.; Miyahara K.; Ueda A.; Asaoka M.; Okazaki M.; Kawate T.; Kuroda M.; Miyagi Y.; Ishikawa T. Differences in drug sensitivity between two-dimensional and three-dimensional culture systems in triple-negative breast cancer cell lines. Biochem. Biophys. Res. Commun. 2020, 533 (3), 268–274. 10.1016/j.bbrc.2020.08.075. 32958246

[ref55] Kim J. B.; Stein R.; O’Hare M. J. Three-dimensional in vitro tissue culture models of breast cancer-- a review. Breast Cancer Res. Treat 2004, 85 (3), 281–291. 10.1023/B:BREA.0000025418.88785.2b. 15111767

[ref56] Sizovs A.; Xue L.; Tolstyka Z. P.; Ingle N. P.; Wu Y.; Cortez M.; Reineke T. M. Poly(trehalose): Sugar-Coated Nanocomplexes Promote Stabilization and Effective Polyplex-Mediated siRNA Delivery. J. Am. Chem. Soc. 2013, 135 (41), 15417–15424. 10.1021/ja404941p. 24083547 PMC4027957

[ref57] Cooperstein M. A.; Canavan H. E. Assessment of cytotoxicity of (N-isopropyl acrylamide) and poly(N-isopropyl acrylamide)-coated surfaces. Biointerphases 2013, 8 (1), 19 10.1186/1559-4106-8-19. 24706136 PMC3979476

[ref58] Ayaani S. P.; Wabitsch M.; Christian M. Adipocyte-Breast Cancer Cell Co-Culture in Transwells. Methods Mol. Biol. 2022, 2508, 59–68. 10.1007/978-1-0716-2376-3_6. 35737233

[ref59] Wang S.; Liu X.; Huang R.; Zheng Y.; Wang N.; Yang B.; Situ H.; Lin Y.; Wang Z. XIAOPI Formula Inhibits Breast Cancer Stem Cells via Suppressing Tumor-Associated Macrophages/C-X-C Motif Chemokine Ligand 1 Pathway. Front Pharmacol 2019, 10, 1371 10.3389/fphar.2019.01371. 31803057 PMC6874098

[ref60] Yancu D.; Viau R.; Sanderson T. Development of an estrogen-dependent breast cancer co-culture model as a tool for studying endocrine disruptors. Toxicol In Vitro 2020, 62, 104658 10.1016/j.tiv.2019.104658. 31629071

[ref61] Khan A. A.; Mudassir J.; Akhtar S.; Murugaiyah V.; Darwis Y. Freeze-Dried Lopinavir-Loaded Nanostructured Lipid Carriers for Enhanced Cellular Uptake and Bioavailability: Statistical Optimization, in Vitro and in Vivo Evaluations. Pharmaceutics 2019, 11 (2), 97 10.3390/pharmaceutics11020097. 30823545 PMC6410192

[ref62] Karakash I.; Vasileska J.; Shalabalija D.; Mihailova L.; Dodov M. G.; Raicki R. S.; Crcarevska M. S. Freeze-drying of nanostructured lipid carriers loaded with Salvia off. extract for Alzheimer’s disease treatment 1. Macedonian Pharmaceutical Bulletin 2020, 66, 219 10.33320/maced.pharm.bull.2020.66.03.109.

[ref63] CAS Database; Potassium Persulfate. Chemical Book, 2023.

[ref64] Kilfoyle B. E.; Sheihet L.; Zhang Z.; Laohoo M.; Kohn J.; Michniak-Kohn B. B. Development of paclitaxel-TyroSpheres for topical skin treatment. J. Controlled Release 2012, 163 (1), 18–24. 10.1016/j.jconrel.2012.06.021. PMC3462247 22732474

[ref65] Kesarwani P.; Tekade R. K.; Jain N. K. Spectrophotometric estimation of paclitaxel. International Journal of Advances in Pharmaceutical Sciences 2011, 2 (1), 29–32.

[ref66] Sugo K.; Ebara M. A simple spectrophotometric evaluation method for the hydrophobic anticancer drug paclitaxel. PeerJ. Analytical Chemistry 2020, 2, e3 10.7717/peerj-achem.3.

[ref67] Wang C.; Yan Q.; Liu H. B.; Zhou X. H.; Xiao S. J. Different EDC/NHS activation mechanisms between PAA and PMAA brushes and the following amidation reactions. Langmuir 2011, 27 (19), 12058–12068. 10.1021/la202267p. 21853994

[ref68] Brugnano J.; McMasters J.; Panitch A. Characterization of endocytic uptake of MK2-inhibitor peptides. J. Pept Sci. 2013, 19 (10), 629–638. 10.1002/psc.2541. 24014473 PMC4034706

[ref69] Carvalho A. F.; Gasperini L.; Ribeiro R. S.; Marques A. P.; Reis R. I. Control of osmotic pressure to improve cell viability in cell-laden tissue engineering constructs. Journal of Tissue Engineering and Regenerative Medicine 2018, 12 (2), e1063–e1067. 10.1002/term.2432. 28342296

[ref70] Ahmad R.; Kaus N. H. M.; Hamid S. Synthesis and Characterization of PLGA-PEG Thymoquinone Nanoparticles and Its Cytotoxicity Effects in Tamoxifen-Resistant Breast Cancer Cells. Adv. Exp. Med. Biol. 2018, 1292, 65–82. 10.1007/5584_2018_302. 30560443

[ref71] Smith M. C.; Gheux A.; Coton M.; Madec S.; Hymery N.; Coton E. In vitro co-culture models to evaluate acute cytotoxicity of individual and combined mycotoxin exposures on Caco-2, THP-1 and HepaRG human cell lines. Chem. Biol. Interact 2018, 281, 51–59. 10.1016/j.cbi.2017.12.004. 29222052

[ref72] Renaud J.; Martinoli M.-G. Development of an Insert Co-culture System of Two Cellular Types in the Absence of Cell-Cell Contact. J. Visualized Exp. 2016, 113, e54356 10.3791/54356. 27500972

