# Supplementary material for: Collagen-Binding Nanoparticles for Paclitaxel Encapsulation and Breast Cancer Treatment
Source: ACS Biomater Sci Eng. 2023 Nov 20;9(12):6805–20. doi: 10.1021/acsbiomaterials.3c01332 (PMC10716849; doi:10.1021/acsbiomaterials.3c01332)
Supplement: Supplementary file 1 — ab3c01332_si_001.pdf [file ab3c01332_si_001.pdf]

Supporting Information

Collagen-binding nanoparticles for paclitaxel encapsulation and breast cancer treatment

Julia Sapienza Passos [1,2] , Luciana B. Lopes [2,#], Alyssa Panitch [1, #, *]

[1] Wallace H. Coulter Department of Biomedical Engineering, Georgia Institute of Technology and Emory University

[2] Department of Pharmacology, Institute of Biomedical Sciences, University of Sao Paulo, Brazil

Table of contents

This document contains five Figures and one Table depicting supporting results, as listed below.

Figure S1. Paclitaxel-loaded (gray bars) and unloaded (black bars) nanostructured lipid carriers stability under heating. The nanocarriers dispersed in water (as obtained) were heated in a mineral oil bath for 4 hours, simulating the synthesis environment of hybrid nanoparticles.

Figure S2. Diameter distribution of pNIPAM and hybrid nanoparticles core (gray lines) and core + shell (black lines) synthesized at 50, 60 and 70 °C.

Table S1. Size, PDI and ζ Potential of optimized nanoparticles.

Figure S3. DLS hydrodynamic diameter temperature sweep from 17.0 – 41.0 °C of paclitaxel-loaded (white circles) and unloaded (black circles) nanoparticles.

Figure S4. Degradation of fluorescently labeled hybrid and pNIPAM nanoparticles dispersed in PBS at pH 7.4 and pH 3.5 over 5 days.

Figure S5. Quantification of collagen secreted by MCF-7 and T-47D cells in culture at days 1 – 7.

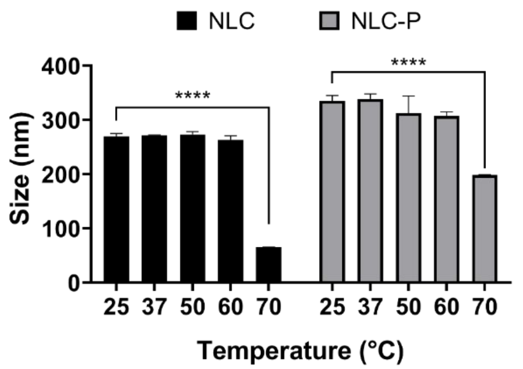

Figure S1. Paclitaxel-loaded (gray bars) and unloaded (black bars) nanostructured lipid carriers stability under heating. The nanocarriers dispersed in water (as obtained) were heated in a mineral oil bath for 4 hours, simulating the synthesis environment of hybrid nanoparticles. **** $p < 0.0001$ compared to 25 °C.

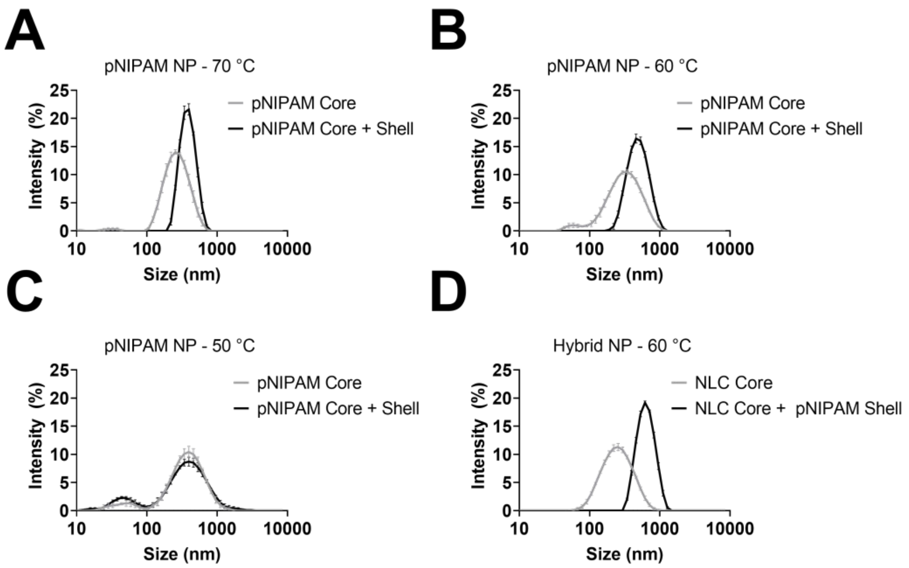

Figure S2. Diameter distribution of pNIPAM and hybrid nanoparticles core (gray lines) and core + shell (black lines) synthesized at 50, 60 and 70 °C. (**A**) pNIPAM NP at 70 °C, (**B**) pNIPAM at 60 °C, (**C**) pNIPAM at 50 °C, and (**D**) Hybrid NP at 60 °C.

Table S1. Size, PDI and ζ Potential of optimized nanoparticles. Data shown represents the mean ± standard error of at least three batches of each formulation.

Formulation	Core	Shell	Size (nm)	PDI	Zeta Potential (mV)
**NLC**	NLC	None	278.5 ± 6.7	0.18 ± 0.03	-11.9 ± 0.5
**NLC-P**	Paclitaxel-loaded NLC	None	341.7 ± 11.2	0.27 ± 0.02	-22.9 ± 0.9
**NLC-P SILY 200%**	Paclitaxel-loaded NLC	None	361.8 ± 12.7	0.22 ± 0.04	-17.9 ± 1.1
**PNIPAM NP**	PNIPAM core (removed by dialysis)	PNIPAM	227.4 ± 7.2	0.19 ± 0.02	-17.8 ± 1.4
**PNIPAM NP-P**	PNIPAM core (removed by dialysis)	PNIPAM	247.2 ± 7.5	0.26 ± 0.03	-24.5 ± 2.3
**PNIPAM NP-P SILY 200%**	PNIPAM core (removed by dialysis)	PNIPAM	261.1 ± 8.3	0.22 ± 0.09	-14.5 ± 0.6
**Hybrid NP**	NLC	PNIPAM	380.4 ± 4.4	0.28 ± 0.05	-15.7 ± 2.0
**Hybrid NP-P**	Paclitaxel-loaded NLC	PNIPAM	383.5 ± 20.2	0.22 ± 0.07	-23.1 ± 1.6
**Hybrid NP-P SILY 200%**	Paclitaxel-loaded NLC	PNIPAM	410.5 ± 10.5	0.23 ± 0.05	-15.0 ± 1.7

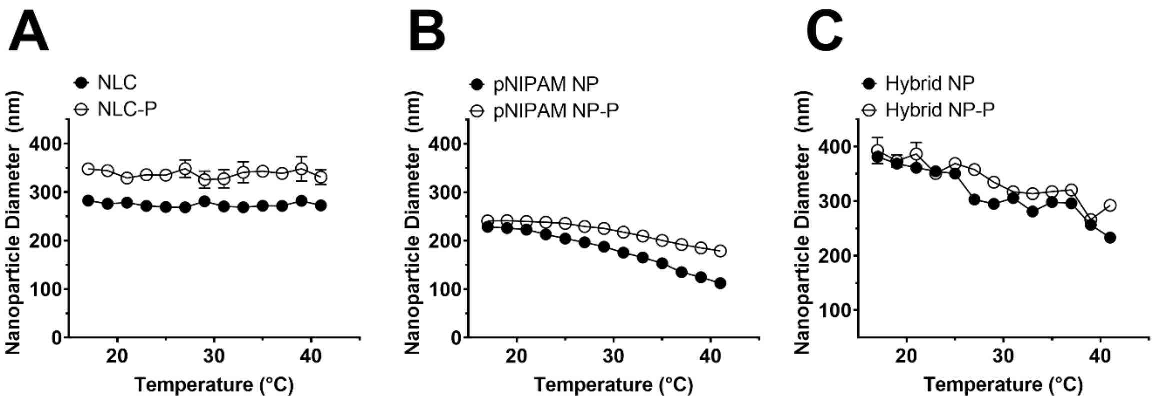

Figure S3. DLS hydrodynamic diameter temperature sweep from 17.0 – 41.0 °C of paclitaxel-loaded (white circles) and unloaded (black circles) nanoparticles. (**A**) Nanostructured Lipid Carriers, (**B**) pNIPAM Nanoparticles and (**C**) Hybrid Nanoparticles.

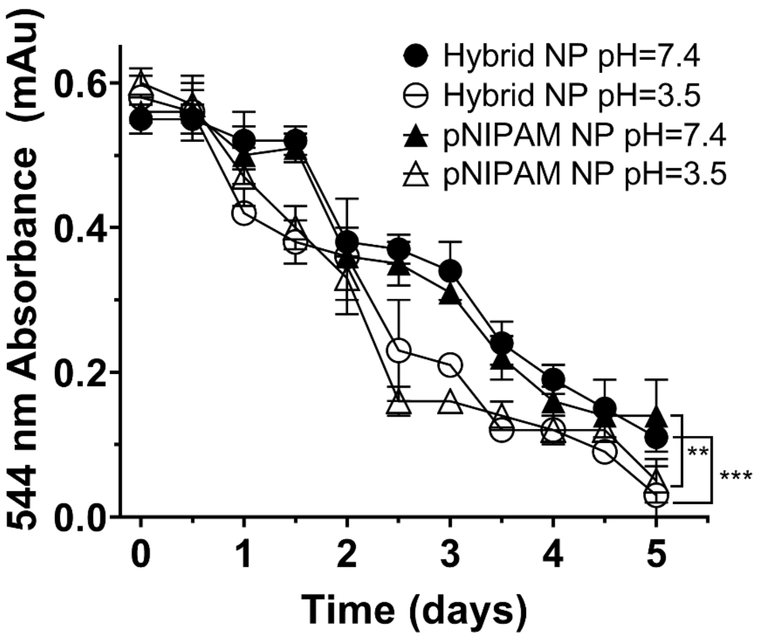

Figure S4. Degradation of fluorescently labeled hybrid and pNIPAM nanoparticles dispersed in PBS at pH 7.4 and pH 3.5 over 5 days. ** $p < 0.01$ and *** $p < 0.001$ compared to the same type of NP at pH 7.4.

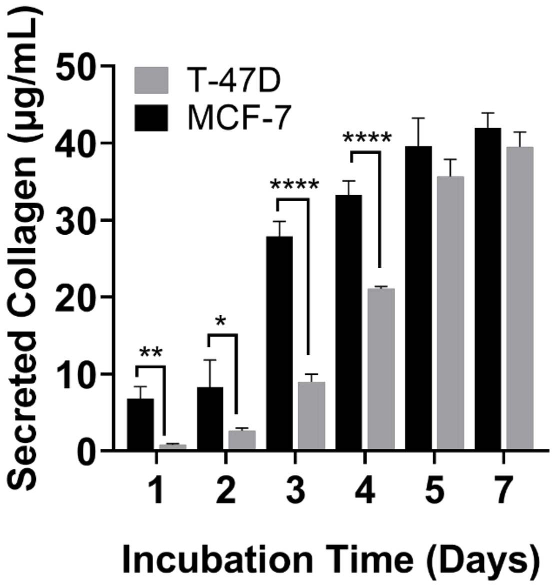

Figure S5. Quantification of collagen secreted by MCF-7 and T-47D cells in culture at days 1 – 7. Data shown as the average ± standard deviation of 6 replicates in 2 independent experiments.
